# Comparing Accuracy and Biases of DNA Metabarcoding, Hybridization Capture, and Metagenomic Sequencing for Quantifying Herbivore Diets

**DOI:** 10.1111/1755-0998.70175

**Published:** 2026-07-16

**Authors:** Charlotte E. Eriksson, Lisa Shipley, Darren A. Clark, Taal Levi

**Affiliations:** ^1^ Department of Fisheries, Wildlife and Conservation Sciences Oregon State University Corvallis Oregon USA; ^2^ School of the Environment Washington State University Pullman Washington USA; ^3^ Oregon Department of Fish and Wildlife La Grande Oregon USA

**Keywords:** diet analysis, feeding trials, herbivores, next‐generation sequencing, relative read abundance, target enrichment

## Abstract

DNA metabarcoding using relative read abundance (RRA) is commonly applied to estimate herbivore diet composition, yet its quantitative accuracy remains uncertain. We assessed taxonomic resolution and quantitative performance of RRA from DNA metabarcoding compared to metagenomic sequencing and hybridization capture, using deer scats from feeding trials and recreated diet samples using plant tissues. All methods recovered plant composition in recreated diets (*R* ^2^ = 0.59–0.82), indicating accurate scaling with biomass in the absence of digestion, with only minor bias from amplicon length in DNA metabarcoding. In contrast, RRA from scat samples performed poorly (*R* ^2^ < 0.01) across all methods largely due to differential plant digestibility. Correcting for digestibility, measured with acid detergent lignin and acid‐insoluble ash, was strongly supported in mixed‐effects models and improved prediction of dietary composition, although species‐level variation remained. For metagenomic sequencing and hybridization capture, we also evaluated Relative Genome Coverage (RGC), a novel relative abundance metric quantifying the proportion of each plant's chloroplast genome covered by mapped reads, normalized for genome length. RGC further improved correlations in recreated diets (*R* ^2^ = 0.82–0.84) and, with hybridization capture, largely overcame digestibility‐related biases in scat samples (*R* ^2^ = 0.57) without correction. When such corrections are infeasible, hybridization capture with uncorrected RGC may achieve higher quantitative accuracy in scat samples. Our results provide practical guidance for improving molecular herbivore diet analysis and highlight the importance of accounting for digestion‐related biases.

## 1 Introduction

DNA metabarcoding has revolutionized diet analysis by enabling the identification of consumed species from animal scats, offering valuable insights into trophic interactions and foraging behaviour. However, the quantitative accuracy of this method remains a significant challenge (Lamb et al. 2019). Typically, relative read abundance (RRA) is used to estimate the proportional representation of dietary items, under the assumption that read counts correlate with consumed biomass (Deagle et al. 2018). In practice, this relationship is often distorted by both biological and technical biases. One major source of bias is differential amplification efficiency, primarily driven by primer‐template mismatches (Piñol et al. 2019). Primers may bind with varying efficiency across taxa, leading to either over‐ or under‐representation of certain diet items. Additional amplification biases can result from differences in GC content (i.e., the percentage of guanine and cytosine bases) and amplicon length, as sequences with high or low GC content or longer lengths tend to amplify less efficiently, further distorting RRA (Shaffer et al. 2025). PCR inhibitors commonly found in plant tissues such as polyphenols and polysaccharides can also reduce overall PCR efficiency and contribute to taxon‐specific biases (Schrader et al. 2012). Other biological factors such as variable digestibility or differences in organelle density (e.g., chloroplasts, mitochondria) further complicate the relationship between read counts and consumed biomass (Deagle et al. 2010; Martin et al. 2022; Vallin et al. 2026; Stapleton et al. 2022). Commonly used plant barcoding regions such as the trnL P6 loop is limited in taxonomic resolution, and often fails to resolve species‐level identity. Incorporating additional loci (e.g., rbcL, matK) can improve taxonomic resolution but introduces new biases and amplification artefacts while also complicating inference about relative abundance across primer sets. Finally, contamination poses a significant concern, as even trace amounts of exogenous DNA can be amplified and misidentified as part of the diet, requiring stringent laboratory protocols and bioinformatic filtering.

To overcome these limitations, PCR‐free methods such as shotgun metagenomic sequencing have been proposed. By sequencing all DNA fragments in a sample, this approach avoids PCR‐related biases and may therefore improve taxonomic resolution and biomass estimation (Bell et al. 2021; Bista et al. 2018). This method can also provide information beyond diet, including host population genetics, microbiomes, and parasites (Chua et al. 2021; Srivathsan et al. 2016). However, metagenomic sequencing remains cost‐prohibitive for large‐scale diet quantification, as the majority of reads originate from non‐target sources such as the host genome and bacteria, requiring deep sequencing to detect dietary components present in low abundance.

Hybridization capture offers a promising intermediate solution. This technique relies on probes designed to enrich specific genetic targets such as plant chloroplast DNA and the removal of off‐target DNA prior to sequencing. By focusing on DNA relevant to diet analysis and avoiding PCR, hybridization capture improves efficiency, reduces off‐target reads, and minimizes amplification bias (Mamanova et al. 2010). Although sequencing depth requirements are lower than for metagenomic sequencing due to the higher target recovery, capture protocols are limited by the costs of probes and reagents as well as labor‐intensive laboratory work relative to DNA metabarcoding (Li et al. 2023).

Despite their potential, non‐PCR based methods face additional challenges. One significant concern is the potential increased risk of false positive identifications, particularly among closely related species that share conserved genomic regions (Bell et al. 2021). Homologous sequences can lead to ambiguous read assignments, as multiple taxa “compete” for alignment to the same regions. Unlike DNA metabarcoding, such errors typically stem from the limited discriminatory power of conserved sequences in reference databases, rather than contamination. As a result, accurate taxonomic identification depends on the availability of comprehensive and well‐curated reference databases, which are essential for minimizing both false positives and false negatives (Chua et al. 2021). These challenges also increase bioinformatic complexity, requiring more advanced pipelines and computational resources compared to established DNA metabarcoding workflows.

**FIGURE 1:**
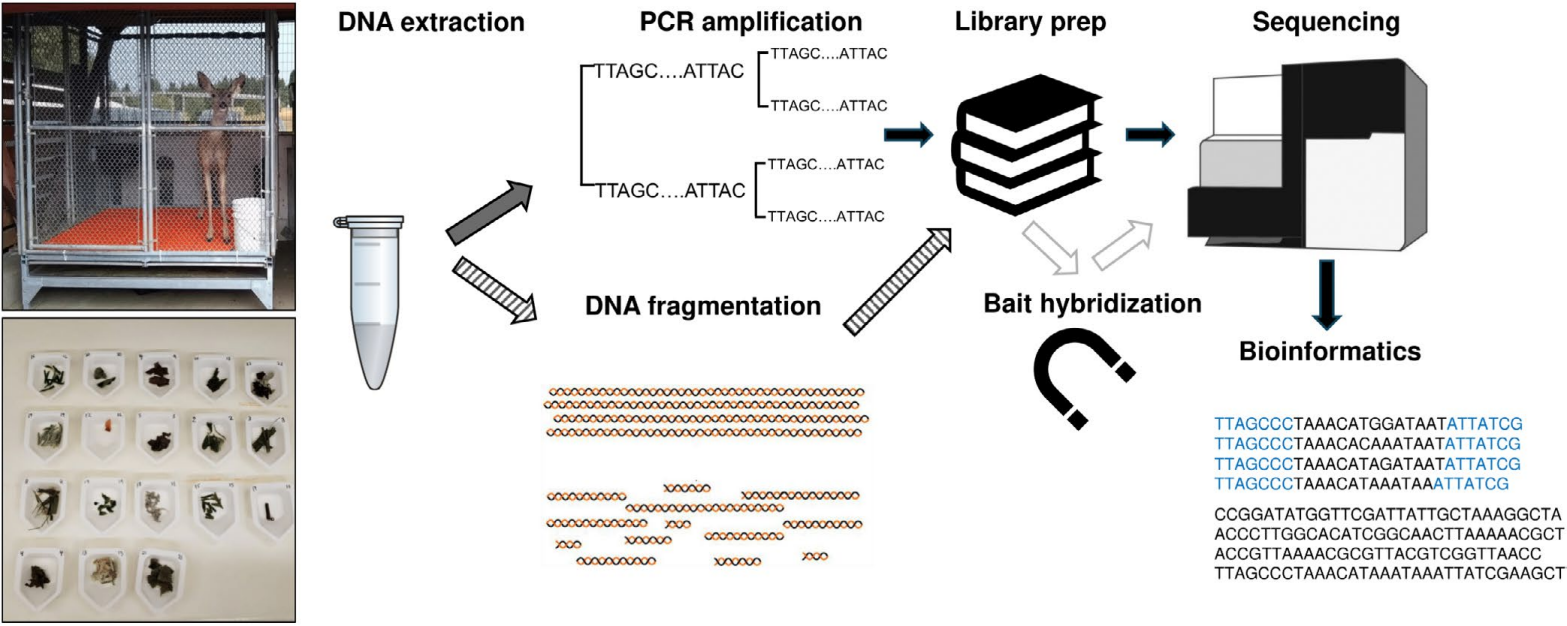
Conceptual diagram of the workflow from samples to taxonomic assignment with DNA metabarcoding, metagenomic sequencing, and hybridization capture. Scat samples from captive mule deer ( *Odocoileus hemionus* ) and white‐tailed deer ( *Odocoileus virginianus* ) in controlled feeding trials (*n* = 26) and recreated diet samples from dried plant tissues (*n* = 26) were extracted, then amplified (DNA metabarcoding) or fragmented (metagenomic sequencing, hybridization capture). Indexes were added to the fragments or PCR products and then sequenced. For hybridization capture, the libraries were first enriched for plant DNA using biotinylated baits which were then separated from the off‐target DNA using magnetic beads. See the Methods section for details on the bioinformatic workflow.

To assess how DNA metabarcoding, metagenomic sequencing, and hybridization capture compare in their ability to recover and quantify dietary components, we conducted controlled feeding trials with deer ( *Odocoileus hemionus* and *O. virginianus* ) fed known diets. For each method, we analysed fecal samples collected from the feeding trials and recreated the same diet compositions using dried plant tissues mixed in the exact proportions consumed to isolate digestion‐related biases from method‐specific biases (Figure 1). We modelled the relationship between the true proportion of each plant species and its method‐specific estimate of relative abundance as a function of plant‐specific nutritional and molecular covariates hypothesized to introduce biased quantification. We also compared two metrics for estimating relative abundance in metagenomic sequencing and hybridization capture: relative read abundance (RRA) and relative genome coverage (RGC). RRA is based on the proportion of sequencing reads mapped to each plant species, whereas RGC reflects the proportion of each species' chloroplast genome covered by sequencing reads (i.e., breadth of coverage). RGC was normalized by genome length to account for potential biases caused by variation in genome size, as longer genomes can attract more reads and potentially inflate RRA‐based abundance estimates. We hypothesized that RGC may provide a more robust estimate of relative abundance under conditions where target enrichment is uneven or sequencing depth is variable, which are common challenges when working with degraded fecal DNA. In such cases, RRA can overestimate the abundance of species with high read pile‐ups at one or few loci, whereas RGC captures how broadly reads are distributed across the genome. By comparing these methods and relative abundance metrics under controlled conditions, our aim was to inform best practices that support more accurate assessments of herbivore diets and foraging behaviour.

## 2 Materials and Methods

### 2.1 Diets

We created nine diets consisting of 2–3 plants and a pelleted diet fed to captive deer in 3‐day trials over a year. The experimental unit was a fecal pellet collected from each deer during a trial. Diets included plants from various functional groups and plant parts including fresh grass, forbs, deciduous shrub/tree leaves, evergreen shrub/tree leaves, cured grass and forbs, stems and fruits (Table 1). Closely related species from the same family were included to assess taxonomic resolution (Table 1). We processed each food item before feeding to ensure uniformity in plant parts and to remove other co‐growing plant species. To maintain sufficient body mass, deer were provided a limited amount (500 or 700 g) of one of three pelleted deer maintenance diets, comprising 33% of their normal daily dry matter intake. These were either milled at Washington State University (WSU) or purchased commercially and consisted of grains, soy, beet pulp, aspen, alfalfa, vitamins and minerals. In our experiments, herbivore pellets were treated as diet items in the same way as plant components.

**TABLE 1:** Diets fed to captive deer. All diets also contained a commercial deer feed pellet consisting of cured grains and forbs with main ingredients including *Medicago sativa* , *Hordeum vulgare* , and *Glycine max* . Three deer were used for each trial except for diet trial #3, which included two, resulting in 26 samples in total and 46 samples × plant species combinations.

Diet	Dates	Plant species	Family	Functional group	Samples
1	26–28 May 2021	*Alopecurus pratensis*	Poaceae	Fresh grass	3
2	2–4 Jun 2021	*Sambucus nigra*	Adoxaceae	Fresh deciduous leaves	3
		*Populus tremuloides*	Salicaceae	Fresh deciduous leaves	3
3	26–28 Jun 2021	*Acer platanoides*	Sapindaceae	Fresh deciduous leaves	2
		*Arrhenatherum elatius*	Poaceae	Fresh grass	2
4	12–14 Jul 2021	*Chamaenerion angustifolium*	Onagraceae	Fresh forb	3
		*Salix lucida*	Salicaceae	Fresh deciduous leaves	3
5	4–6 Dec 2021	*Malus domestica*	Rosaceae	Fresh fruit	3
6	9–11 Feb 2022	*Phleum pratense*	Poaceae	Cured grass	3
		*Thuja plicata*	Cupressaceae	Fresh evergreen leaves	3
7	8–10 Mar 2022	*Salix lucida*	Salicaceae	Fresh deciduous stem	3
8	11–13 Apr 2022	*Artemisia tridentata*	Asteraceae	Fresh evergreen leaves	3
		*Brassica oleracea*	Brassicaceae	Fresh forb	3
9	9–12 Jul 2022	*Rubus pacificus*	Rosaceae	Fresh deciduous leaves	3
		*Fragaria × ananassa*	Rosaceae	Fresh form	3
		*Rosa canina*	Rosaceae	Fresh deciduous leaves	3

### 2.2 Animals

Three deer were used per trial except for diet trial #3 (*n* = 2). Seven individual deer ( *O. hemionus* , *O. virginianus* ) were used across the trials. All deer were female, aged 6–14 years old, and accustomed to the digestion crates and feeding trials from previous experiments. Animal procedures were approved by IACUC protocol #6727.

### 2.3 Feeding Trials

Deer were maintained on the pellet diet for ≥ 2 weeks before each trial, with limited access to pasture grass and alfalfa hay. On the first day of each trial, deer were placed singly in 2.5 × 2.5 m digestion crates with a mesh floor that allowed feces to fall through and collect on a screen and urine to be funnelled to a container underneath (Figure 1). Each morning of the trial, we weighed out the pelleted diet (500 or 700 g), then weighed enough of the 1–3 plant foods for ad libitum intake. One fresh sample of each food (plants and pellets) was weighed, dried at 100°C for 24 h, and reweighed to calculate percent dry matter to correct for water in the food. Two additional samples of each food item were frozen: one for nutritional analyses (later freeze‐dried and ground), and one for DNA analyses. Each food item was offered simultaneously in a separate food container. The following morning, we collected, separated, and weighed (to the nearest 0.1 g) all food remaining within the digestion crate, and took a sample to correct for dry matter. Fecal pellets were collected daily from the floor of the crate or on the screen below using nitrile gloves. About 20 fecal pellets per animal and feeding trial were placed in a sterile bag each day and stored in a −20°C freezer until DNA extraction. At least 2 weeks passed between trials and deer were rotated.

### 2.4 Diet Composition

Daily intake of each food item was calculated by subtracting dry mass remaining each morning from dry mass offered the previous morning. The percent composition of each food item consumed each day was calculated as the dry mass consumed of that food item relative to total dry mass of all food items consumed, and composition was averaged across the 3‐day trial.

### 2.5 Fiber Analysis of Diet Items

Fiber components of plant cells influence how much of the plant is digested by the herbivore, thus how much of the plant passes into the feces. To quantify variation in digestibility among food items, we first measured the components of plant fiber in each item using sequential detergent analysis at WSU, including neutral detergent fiber (NDF, %), acid detergent fiber (ADF, %), acid detergent lignin (ADL, %), and acid insoluble ash (AIA, %) with sodium sulfite for shrubs and forbs; Goering and van Soest 1970; Ankom Fiber Analyser 200/220, Ankom Technology, Fairport NY, USA. We corrected these plant fiber metrics using eqs. (1–3) in Cook et al. (2022). We then used the corrected plant fiber metrics to estimate deer‐specific dry matter digestibility (DMD, %) based on the equation in Robbins et al. (1987, 1610). Protein reduction from tannin binding was assumed to be 0% for these calculations.

### 2.6 DNA Extraction

We dried 3 fecal pellets from each sample collected on feeding trial Day 3 (*n* = 26 samples in total) for 3 h at 65°C. Once dried, we used a razor blade and tweezers to scrape three small pieces (20 mg total weight) from each of the three fecal pellets into a 2 mL tube and added five 2.3 mm Zirconia/Silica beads (Biospec Products). We soaked the tools in 50% bleach, rinsed with deionized water, and placed under UV light for 2 min between samples to prevent contamination. The samples were homogenized for 2 min at 3200 rpm using a bead beater followed by DNA extraction using the Qiagen Plant Mini Kit (Qiagen) as per the manufacturer's protocol except that we eluted in 60 μL twice for a total volume of 120 μL. Frozen plant tissues were dried as described above (*n* = 16 plant tissues and 3 commercial pellet types). We prepared plant tissue mixes in the same proportions as was consumed by each individual deer to isolate the effects of digestion on relative abundance (hereafter ‘recreated diet samples’ *n* = 26). For example, if a deer consumed three plant species in the following proportions: 0.79, 0.09 and 0.11, then 79 mg, 9 mg and 11 mg of dried plant matter was added from each plant species respectively (total weight 100 mg). The plant mixture was then homogenized as described above, and 20 mg of the homogenized plant matter was placed in a new 1.7 mL tube followed by DNA extraction. Each individual feeding trial plant species and feed pellets were also extracted individually as described above. We included one negative control per extraction batch to monitor for contamination. We assessed DNA purity using a Nanodrop Spectrophotometer and total DNA concentrations using a Qubit 2.0 fluorometer (Life Technologies) with the AccuGreen High Sensitivity dsDNA Quantitation Kit (Biotium). Blanks with DNA concentrations that were too low to be measured were not analysed further.

### 2.7 DNA Metabarcoding

We amplified the trnL P6 loop region using the universal g‐h primers (g‐F: GGGCAATCCTGAGCCAA and h‐R: CCATYGAGTCTCTGCACCTATC) (Taberlet et al. 2007) with unique and identical 8 base pair tags on the 5′ end of both primers. We performed 3 PCR replicates per sample and included 3 no‐template controls per 96‐well plate to monitor for cross‐contamination. Each PCR reaction consisted of 10 μL Amplitaq Gold Master mix (ThermoFisher Scientific), 3 μL water, 6 μL of each primer (0.3 μM final concentration), and 1 μL DNA template. After a 10 min initial denaturation at 95°C, the cycling conditions were 95°C for 30 s, 55°C for 30 s, 72°C for 60 s for 35 cycles followed by a 7 min final extension at 72°C. All DNA extractions and PCR plates were prepared within a pre‐PCR laboratory. The PCR products were quantified using a fluorescence microplate reader with the AccuBlue dsDNA High Sensitivity Quantitation Kit (Biotium), normalized accordingly, and pooled. Illumina sequencing libraries were constructed using the NEBNext Ultra II Library Prep Kit (New England Biolabs), purified using Mag‐Bind TotalPure NGS (Omega Bio‐tek), quantified using AccuGreen Broad Range dsDNA quantitation kit (Biotium), and finally normalized and pooled. We combined our trnL library with other projects on a P2 300 cycle 2 × 150 bp NextSeq 2000 sequencing run at the Center for Quantitative Life Sciences (CQLS), Oregon State University, USA.

### 2.8 Metagenomic Sequencing

The deer scat and recreated diet extracts were submitted for library preparation and sequencing at the Genomics and Cell Characterization Core Facility (GC3F), University of Oregon, USA. 10 ng of total DNA for each sample was transferred to a 384 well plate and then dried using a Speed‐Vac. Illumina tagmentation beads were added to a 10.5 μL custom tagmentation buffer (20 mM Tris (pH 7.6), 20 mM MgCl_2_ and 20% (v/v) dimethylformamide (DMF)), and incubated for 15 min at 55°C. Beads were collected on the side of the wells using a magnetic plate holder and 9 μL of supernatant was removed. 1 μL of 10 μM barcoded Illumina primer, 3 μL nuclease free water and 5 μL Q5 HotStart Master Mix (New England Biolabs) were added to each sample. Samples were heated to 68°C for 3 min to release from beads, then to 98°C for 3 min to denature the samples. Samples were amplified with 10 cycles of 98°C for 45 s, 62°C for 30 s, and 68°C for 2 min, followed by a 1 min final extension at 68°C. Amplified samples were diluted by adding 20 μL nuclease free water per sample. 2 μL per sample were pooled and bead cleaned twice with a 0.8× ratio of Mag‐Bind Total Pure NGS beads (Omega Bio‐Tek). Pooled samples were run on a MiSeq Nano flow cell and read counts obtained for each sample. Samples were re‐pooled based on the output of the MiSeq run, cleaned as above and sequenced on a single lane of a NovaSeq S4 flow cell.

### 2.9 Hybridization Capture

#### 2.9.1 Bait Design

We targeted five DNA barcode regions in the chloroplast genome: rbcL, matK, rpoB, and the spacer regions trnH‐psbA and trnL‐trnF. We downloaded all available embryophyte sequences for the selected gene and spacer regions in GenBank and selected one representative from each family to avoid taxonomic over‐representation in the probe pool, resulting in 762 targets. In total, 135,662 baits were initially designed using 80 nt probes and 3× tiling by Daicel‐Arbor Biosciences. We then filtered out any baits that matched the mule deer (GCA_020976825.1_BYU_Ohem_2021) or white‐tailed deer genomes (GCA_002102435.1_Ovir.te_1.0) or bacteria using BLAST and a proprietary hybridization Tm estimator, resulting in a final set of 123,196 baits.

#### 2.9.2 Library Preparation

A total of 300 ng of each plant tissue and recreated diet sample extracts, and 1000 ng of each scat sample extract, were sheared to a size distribution centered at ~300 bp using a sonicator (Diagenode Bioruptor Pico). Sizes were checked before and after sonication for a subset of samples using a High Sensitivity D5000 Tapestation (Agilent Technologies). Illumina libraries were prepared using the NEBNext Ultra II Library Preparation Kit with unique dual index adaptors (New England Biolabs) without size selection. Post adaptor ligation we amplified the libraries to detectable concentrations using 5 PCR cycles. We followed the manufacturer's instructions with the exception of the final bead cleanup where we eluted in 16 μL 0.1× TE instead of 33 μL using the Mag‐Bind Total Pure NGS beads (Omega Bio‐Tek). We quantified the library concentrations using a fluorescence microplate reader and the AccuBlue HS dsDNA Quantitation kit (Biotium).

#### 2.9.3 Probe Enrichment and Sequencing

The plant tissues and recreated diet sample libraries were pooled in approximately equal post‐library prep DNA concentrations (11–13 libraries per pool, respectively). Scat sample libraries were pooled based on the proportion of plant DNA found in each library as estimated from the metagenomic sequencing output. This was done to ensure libraries contributed similar amounts of target DNA and so that samples with the least amount of plant DNA were allocated to smaller pools. This resulted in 5 pools with 1 pool of 4 libraries (~0.1–0.4 ng of target DNA), 2 pools of 5 libraries in each (~0.4–1 ng and 1–3 ng, respectively) and 2 pools of six libraries (~3–6 ng and 8–56 ng, respectively). For the hybridization enrichment we followed the myBaits Standard protocol (Daicel‐Arbor Biosciences) for the plant tissue and recreated diet sample pools with a 60°C incubation for 24 h. For the scat sample pools, we followed the High Sensitivity protocol with an incubation temperature of 60°C for 48 h. Following hybridization enrichment and amplification, we purified the pools using the Mag‐Bind Total Pure NGS beads and quantified the DNA concentrations using the AccuGreen Broad Range dsDNA Quantitation Kit. The pools were then normalized and pooled before sent for 150 bp paired‐end sequencing on an Illumina NextSeq 2000 P2 run at CQLS.

### 2.10 Bioinformatic Analysis

#### 2.10.1 DNA Metabarcoding

We used PEAR (Zhang et al. 2013) to pair the raw sequence reads and demultiplexed the paired reads using the unique 8 bp‐index sequences with a custom shell script. Unique reads from each sample replicate were counted, clustered at 100%, and taxonomically assigned using BLAST (www.ncbi.nlm.nih.gov/blast) within the R package LocaTT (Goodwin et al. 2026) against all trnL plant sequences in GenBank. If only one species matched to 100% and was locally present in the area, we assigned it to species level. If multiple locally occurring species matched to 100%, or if the match was < 99%, the sequence was assigned to ‘Genus’ or ‘Family’ level. We removed sequences that made up < 0.5% of the total number of sequences for a sample and sample replicates with fewer than 500 reads in total. Sequences present in only 1 out of 3 replicates post‐filtering were removed. No sequences occurring in the extraction blanks or no‐template controls made the filtering cut‐offs.

#### 2.10.2 Metagenomic and Hybridization Capture Samples

We trimmed adapters, removed low quality reads (Phred < 30) and filtered out sequences < 50 bp using fastp (‐q 30 ‐l 50) (Chen et al. 2018) and assessed post‐processing quality with FastQC v. 0.12.1 (https://www.bioinformatics.babraham.ac.uk/projects/fastqc/). We first used Kraken2 (Wood et al. 2019) to quantify the proportion of reads from the scat samples that was derived from plants. Kraken2 uses an alignment free algorithm to classify short reads based on comparing short substrings (*k*‐mers) from sequence reads to a reference database. If a *k*‐mer is shared between two species the lowest common ancestor is provided. To do this, we created a custom database compiled from the RefSeq libraries archaea, bacteria, fungi, human, plant, protozoa, viral as well as the NCBI nucleotide database (nt) and UniVec database (e.g., adapters, linkers, and primers) (accessed May 2024) as well as the genomes of white‐tailed deer and mule deer. We used default settings and the ‐paired function to concatenate the forward and reverse reads. We further used Kraken2 for taxonomic assignment and diet quantification with a small library consisting of the chloroplast genomes of the 15 feeding trial plants and 9 commercial pellet ingredient species (Table S1) with a confidence score of 0.5 for the metagenomic samples and 0.05 for the hybridization capture samples to minimize false positive assignments. We used Bracken (Lu et al. 2017) to estimate relative read abundance of species from the Kraken2 taxonomic assignments and filtered out species found at less than 0.1% of the total reads per sample for metagenomic sequencing, and 1% for hybridization capture. See Table S2 for a comparison of Kraken2 confidence scores and read filtering thresholds.

Read mapping‐based approaches using aligners such as bwa or minimap2 are slower but considered more sensitive than Kraken2 (Lu et al. 2022). We therefore used minimap2 (Li 2018) to align the trimmed reads to the chloroplast reference library (Table S1). We used Samtools (Li and Durbin 2009) to filter out unmapped, secondary, and supplementary reads, and to retain only properly paired reads with high mapping quality (‐F 2308 ‐f 0 × 2 ‐q 48). Reads from the hybridization capture data were also deduplicated. These stringent filters retain high‐confidence primary alignments and reduce inflation of coverage stemming from conserved regions shared among closely related taxa. To evaluate the potential impact of competitive mapping among closely related taxa, we used hybridization capture data generated from the single‐species plant tissue samples and compared the proportion of the genome covered when aligning to a combined reference library containing all taxa versus mapping reads to each species' chloroplast genome independently. We calculated per base coverage using the genomecov function in BEDTools (Quinlan and Hall 2010). Since hybridization capture can often recover full chloroplast genomes from off‐target sequences (Waycott et al. 2021), we mapped our reads to the full chloroplast genomes to ensure we used as much of the sequencing data as possible. We evaluated taxonomic assignment using both Kraken2 and read mapping independently. Because each method has different strengths and limitations, we additionally assessed a combined strategy retaining taxa identified by both approaches (see Results). We estimated false positives and negatives using these methods based on the recreated diet samples because deer had limited pasture access before the feeding trials began and trace amounts of DNA remained within their digestive systems when we collected the scats. A false negative was defined as failure to detect a plant species known to be included in a recreated diet sample, whereas a false positive was defined as detection of a plant species not included in that sample.

### 2.11 Chloroplast Copy Number

The trnL‐trnF intergenic spacer is located in a single copy region of the chloroplast genome, making it a suitable target for comparing relative chloroplast copy number across plant species in the feeding trial using the trnL g–h primers. The plant tissue extracts were diluted to 0.05 ng/μL and prepared in singleplex digital PCR reactions using the QuantStudio Absolute Q Digital PCR System (Applied Biosystems). 1 μL of the diluted DNA extracts was mixed with 2 μL Absolute Q DNA Digital PCR Master Mix, 0.4 μL SYBR Green I Nucleic Acid Gel Stain, 0.2 μL of the forward and reverse primers (200 nM final concentration), and 6.2 μL water. After vortexing, 9 μL of the reagent mixture was loaded to MAP16 digital PCR plates followed by 15 μL of isolation buffer following the manufacturer's protocol. Each sample was run in duplicate, and we included two no‐template controls per plate. After a 10 min activation step at 96°C, the cycling conditions were 96°C for 5 s, and annealing at 60°C for 45 s for 40 cycles. These measurements reflect chloroplast copy number per 0.05 ng of total DNA, which will also contain nuclear DNA. As a result, values are influenced by both chloroplast abundance and nuclear genome size, with larger genomes potentially contributing proportionally less chloroplast DNA. To evaluate whether variation in nuclear genome size biased our chloroplast copy number estimates, we performed hybridization capture on single‐tissue samples and assessed the number of chloroplast reads recovered. The read counts were significantly correlated with the digital PCR‐based chloroplast copy number estimates (Spearman's *ρ* = 0.71, *p* = 0.003), suggesting that nuclear genome size did not substantially confound our measurements.

### 2.12 Correlation Between Plant Consumption or Composition and Molecular Estimates

We first assessed the correlation between true biomass (the proportion of dry mass that the deer consumed, or that was included in the recreated diet samples) and the relative abundance estimated by molecular methods using RRA for all three methods and RGC for metagenomic sequencing and hybridization capture. For DNA metabarcoding, RRA was defined as the number of reads per plant species divided by the total number of reads in the sample. For hybridization capture and metagenomic sequencing, it was defined as the number of reads that mapped to each species relative to the total number of mapped reads. The RGC calculation required additional steps. We calculated the proportion of the genome covered (breadth of coverage) by dividing the number of bases with mapped reads (per base coverage) by the total length of the species' chloroplast genome (i.e., a normalized coverage count). RGC was then calculated as the normalized count per species divided by the sum of normalized counts across all species in the sample. To assess the direction and consistency of species‐specific biases (i.e., whether some taxa were consistently over‐ or underrepresented), we further calculated the raw difference between relative abundance and true biomass for each species. To quantify overall error across methods, we also calculated the Mean Absolute Error (MAE).

### 2.13 Statistical Models

To identify factors introducing bias in diet quantification, we used the lme4 package (Bates et al. 2015) to fit linear mixed‐effects models using a random intercept for each plant species to account for structure in the residuals due to repeated measures of species identity across samples. Separate models were constructed for each molecular method, with the logit‐transformed RRA or RGC as the response variable for the non‐amplicon approaches, and for both sample types (deer scat and recreated diets). The *Composition* models included only the logit‐transformed proportion of that plant's biomass consumed by deer or used to construct the recreated diet samples. Subsequent models built on these base models. The *Digestion* models additionally included covariates to account for digestibility‐related biases. To determine which covariates to include in the digestion model, we compared candidate submodels using Akaike's Information Criterion (AIC). Our candidate submodels included two approaches. First, we considered digestible dry matter (%DMD). However, because %DMD is a derived metric with limited availability for other herbivores, we also evaluated models using the directly measured plant fiber components obtained via sequential detergent analysis. These included %ADL (indigestible lignin and cutin), %AIA (indigestible minerals), both ADL + AIA, and %NDF (all cell wall components), which we did not include in models with ADL or AIA because these fiber metrics are used to calculate NDF. We hypothesized that DMD would best reflect digestibility but that ADL would be a good proxy because it is highly anticorrelated with digestibility (*r* = −0.91 for our plant species). The most consistently supported submodel based on AIC was then carried forward into the *Digestion* model. The *Molecular* model included the percent GC content (based on G and C bases of the trnL amplicon or chloroplast genome), the length of the amplicon (metabarcoding) or chloroplast genome (metagenomics and hybridization capture), and the chloroplast copy number. Primer mismatch was excluded due to low variability and minimal influence among the feeding trial species. Only two feeding trial species showed minor mismatches with the trnL primers: *Acer platanoides* and *Thuja plicata* had a single mismatch in the reverse primer located three bases from the 5′ end. *T. plicata* also had a single mismatch at the 5′ end of the forward primer. Because mismatches near the 5′ end have a much smaller effect on PCR efficiency than those near the 3′ end, these mismatches are unlikely to substantially reduce amplification or bias results. All other species had perfect matches to both primers. The *Full* model included all covariates. To assess whether phylogenetic relatedness within the reference library would introduce bias in relative abundance estimates from the read‐mapping approaches, we extended the top‐performing models to include a fixed effect representing the number of species in the reference library belonging to the same plant family as each focal species. A negative coefficient for this term would indicate that competitive mapping among closely related taxa is biasing inference on relative abundance downward. All digestion and molecular covariates were standardized (mean = 0, SD = 1) to facilitate effect size comparisons. We calculated the effect sizes for all models and additionally plotted the marginal effects with partial residuals overlaid to facilitate interpretation of the effects of each covariate. We used marginal and conditional *R* ^2^ denoted as *R* ^2^m and *R* ^2^c, respectively, to estimate variance explained by fixed effects only or including the species random effect. Reads from *Fragaria × ananassa* and *Rubus pacificus*, which were included in the same feeding trial, could not be distinguished using the trnL primers. As a result, they were pooled for the analysis of the relationship between RRA and known plant biomass and excluded from species‐specific error estimation and bias modelling within the DNA metabarcoding dataset. All statistical analyses were conducted in R version 4.4.1 (R Core Team 2024).

## 3 Results

### 3.1 Feeding Trials

The average proportion of each plant species consumed by deer in the feeding trials varied considerably (mean 0.18 ± 0.13 SD), ranging from highly consumed species such as *Acer platanoides* (0.498 ± 0.034) and *Salix lucida* (0.381 ± 0.127) to lightly consumed plants such as *Arrhenatherum elatius* (0.040 ± 0.030). The average proportion of commercial pellets consumed by the deer was 0.686 (SD = 0.177), ranging from 0.411 to 0.986. Detailed composition of each feeding trial sample is shown in Figure S1.

### 3.2 Overview/Sequencing Success

After quality filtering, DNA metabarcoding yielded 13.9 million reads ranging from 14,383 to 222,064 per sample replicate (mean 75,059.36 ± 35,491.21 SD) including all sample types (single plant tissues, plant tissue mixes and deer scats). None of the extraction blanks or no‐template controls passed quality filtering. One scat sample was removed from further analysis because it failed library prep for both metagenomic sequencing and hybridization capture. One recreated diet sample failed metabarcoding. The remaining samples were retained across all three methods. Post‐trimming, metagenomic sequencing yielded 0.94 to 55 million reads per sample (mean 42.0 million ±8.7 SD). On average, 4.3% ± 2.8% of the scat sample reads came from plants. In comparison, hybridization capture resulted in 4.27 million reads (±5.29 SD) per sample on average across all sample types after trimming (ranging from 0.28 to 31.1 million reads). The proportion of plant‐derived reads in the scat samples increased to on average 95% (±13.4 SD) per sample. The five gene or spacer region targets were successfully recovered in all plant tissue samples with one exception (trnH‐psbA was missing in *A. elatius* ), and represented on average 3.2% (±2% SD) of the reads per tissue sample, indicating consistent enrichment of the targeted regions among the different plant species as well as substantial nontarget enrichment of additional plant DNA. The average proportion of chloroplast genome recovered per species when aligning to individual genomes was 88% (±8% SD). This declined to 76% (±15% SD) when using a combined reference library indicating some competitive read mapping. The largest difference was seen in *Poaceae* species where the increase in genome proportion was 25%–27% per species when mapped individually. In comparison, species without multiple family representatives in the reference library such as *Chamaenerion angustifolium* (*Onagraceae*) and *Thuja plicata* (*Cupressaceae*) had a 0% or 2% increase, respectively, using individual mapping.

### 3.3 Taxonomic Resolution and Accuracy

DNA metabarcoding identified 10 of the 15 feeding trial plants to species level because *Fragaria × ananassa* and *Rubus pacificus* shared identical trnL sequences as did *Phleum pratense* and *Alopecurus pratensis* . These species would therefore have only been assigned to Family level without prior diet knowledge. *Sambucus nigra* and *Sambucus racemosa* could also not be distinguished using the trnL locus and both occur locally so would be assigned to genus. In contrast, metagenomic sequencing and hybridization capture achieved species‐level resolution for all feeding trial plants.

In total, we had 46 sample × plant species combinations in our feeding trial dataset (i.e., each plant species known to be present in each recreated diet sample). The read mapping method resulted in 1 false negative with metagenomic sequencing whereas hybridization capture detected all 46 species combinations in the recreated diet samples. However, the read mapping method resulted in many false positives that could not be effectively filtered out using a minimum threshold of either percent genome coverage as recommended by Ji et al. (2020) or relative read abundance without also removing many true positives (Table S3).

Taxonomic classification with Kraken2, using a confidence threshold of 0.05 (metagenomic) or 0.5 (hybridization capture) and a minimum read abundance threshold (0.1% for metagenomic sequencing; 1% for hybridization capture), better distinguished closely related species and reduced false positives (Table S2). However, the relative genome coverage (RGC) from the read mapping method correlated more strongly with true biomass (see below) compared to relative read abundance from Kraken2 and Bracken (*R* ^2^ = 0.63 and 0.30, respectively; both *p* < 0.001). The most accurate approach, in terms of both taxonomic assignment and relative abundance, was achieved by intersecting Kraken2 and read mapping results (similar to Garrido‐Sanz et al. 2022): retaining only taxa identified by both methods, with abundance estimated from read mapping and RGC. Using this combined strategy, metagenomic sequencing detected 42 of 46 possible species × sample combinations (91.3%) with no false positives. Hybridization capture initially detected all combinations, but stricter filtering (≥ 1% read abundance) was needed to reduce false positives, resulting in 40 of 46 combinations detected (87%), with 6 false negatives and 1 false positive. By comparison, one recreated diet sample failed to amplify during DNA metabarcoding, and two Rosaceae species could not be distinguished due to identical trnL sequences, reducing the total possible combinations to 41. After filtering out taxa below 0.5% of reads per sample, DNA metabarcoding recovered 34 of 41 combinations (82.9%), with 7 false negatives and 2 false positives. *Malus domestica* fruit was missed in all three recreated diet samples across all methods due to poor homogenization of the dried fruit during the extraction process, but was successfully detected in scat samples.

### 3.4 Recreated Diet Sample Correlations

**FIGURE 2:**
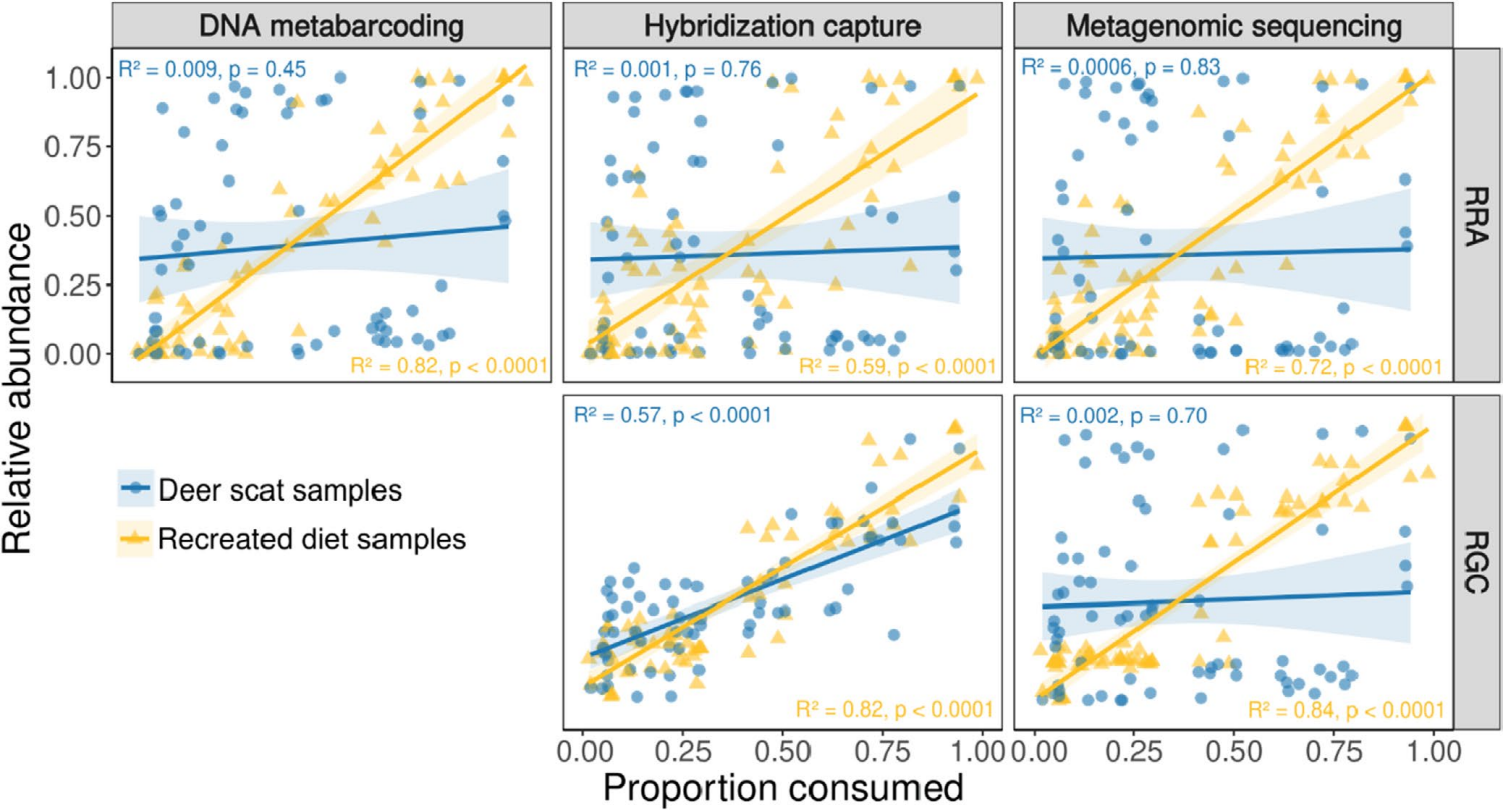
Relationship between the known biomass of plant species and relative abundance estimates in scats of captive mule deer ( *Odocoileus hemionus* ) and white‐tailed deer ( *Odocoileus virginianus* ) from controlled feeding trials and recreated diet samples (plant tissue mixes), using three sequencing methods. Relative abundance was measured as relative read abundance (RRA) for all methods, and as relative genome coverage (RGC) for metagenomic sequencing and hybridization capture. Each point represents a plant species detected in a sample, and lines show linear regressions.

**FIGURE 3:**
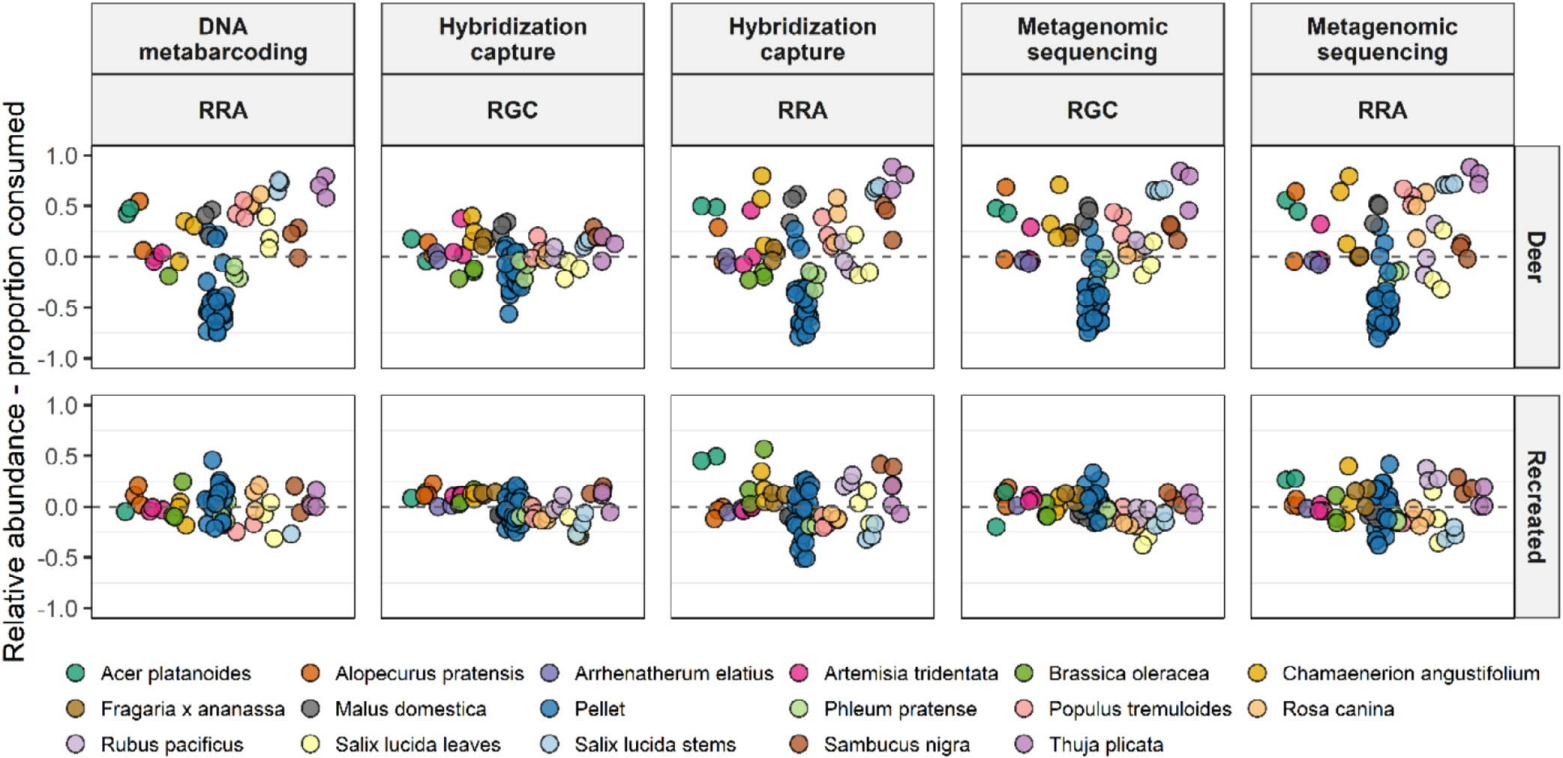
Plant species‐specific bias in relative abundance estimates measured using relative read abundance (RRA) and relative genome coverage (RGC). Each point represents the difference between observed relative abundance and the known biomass for a given plant species in a sample. Positive values indicate overestimation, and negative values indicate underestimation. The dashed horizontal line at zero represents perfect agreement between observed and true values.

There was a significant correlation with high variance explained between RRA and known biomass of plants in the recreated diet samples across all three methods: DNA metabarcoding (*R* ^2^ = 0.82, *p* < 0.0001), metagenomic sequencing (*R* ^2^ = 0.72, *p* < 0.0001), and hybridization capture (*R* ^2^ = 0.59, *p* = 0.0001). When RGC was used for the two non‐amplicon methods, correlations with true plant composition proportions improved (metagenomic sequencing: *R* ^2^ = 0.84, hybridization capture: *R* ^2^ = 0.82, both *p* < 0.0001) (Figure 2). Mean absolute error was lowest for metagenomic sequencing with RGC (mean = 0.097, SD = 0.073), followed by hybridization capture (RGC mean = 0.102, SD = 0.064), and then DNA metabarcoding (RRA mean = 0.114, SD = 0.10). At the species‐level, errors in recreated samples clustered around zero across methods (Figure 3).

### 3.5 Deer Scat Sample Correlations

In contrast, the proportion of plants consumed explained little of the variance in RRA in deer scat samples: DNA metabarcoding (*R* ^2^ = 0.009, *p* = 0.45), metagenomic sequencing (*R* ^2^ = 0.001, *p* = 0.83), and hybridization capture (*R* ^2^ = 0.001, *p* = 0.76). When RGC was used, metagenomic results remained poorly correlated (*R* ^2^ = 0.002, *p* = 0.70), but hybridization capture became significant with moderately high variance explained (*R* ^2^ = 0.57, *p* = 0.0001) (Figure 2). Mean absolute error was higher and more variable for scat samples compared to recreated samples. Hybridization capture using RGC produced the lowest error for scat samples (mean = 0.14, SD = 0.11) whereas RRA for the same method showed substantially higher error (mean = 0.37, SD = 0.24), similar to metagenomic sequencing (RGC mean = 0.35, SD = 0.24; RRA mean = 0.40, SD = 0.26) and DNA metabarcoding (RRA mean = 0.40, SD = 0.22). Species‐level errors were more variable, ranging from −0.72 to 0.81 for DNA metabarcoding and −0.72 to 0.87 for metagenomics and RGC. Both methods consistently overestimated *Thuja plicata* (mean 0.7 ± 0.1 SD for DNA metabarcoding and 0.72 ± 0.23 for metagenomics) and *Salix lucida* stems (mean 0.7 ± 0.03 and 0.66 ± 0.05, respectively). Hybridization capture produced smaller and more evenly distributed errors across plant species when using RGC (range: −0.54 to 0.36) (Figure 3). *Brassica oleracea* was undetected in deer scat by metagenomic sequencing and detected in only one of three DNA metabarcoding samples, despite moderate consumption levels (Figure S1). In contrast, hybridization capture detected *B. oleracea* in all three scat samples, although it was slightly underestimated (mean error: −0.15 ± 0.04). All methods detected *B. oleracea* in recreated diet samples, suggesting its underrepresentation in scat reflected digestion effects rather than bioinformatic limitations.

### 3.6 Statistical Models

The digestion submodel strongly supported inclusion of acid detergent lignin (ADL) and acid‐insoluble ash (AIA), both of which were in the top‐ranked model for RRA when using DNA metabarcoding (Table 2), metagenomic sequencing, and hybridization capture data. Contrary to expectations, DMD was substantially less supported in all models (delta AIC > 9). We therefore moved ADL and AIA forward to the *Digestion* model.

**TABLE 2:** Model selection for the digestion submodel based on RRA from deer diet analysis. All models included logit‐transformed plant composition and the specified covariates. All models supported the inclusion of ADL (lignin and cutin) and AIA (acid‐insoluble ash). ADL and AIA moved forward into the *Digestion* model in the second stage of model selection.

Analysis	Model	AIC	dAIC
DNA metabarcoding	**ADL + AIA**	**279.75**	**0.00**
	ADL	285.69	5.94
	DMD	289.13	9.38
	NDF	291.69	11.94
Metagenomic sequencing	**ADL + AIA**	**315.96**	**0.00**
	ADL	321.97	6.01
	DMD	326.40	10.44
	NDF	327.75	11.78
Hybridization capture	**ADL + AIA**	**302.02**	**0.00**
	ADL	306.45	4.43
	NDF	311.17	9.15
	DMD	311.27	9.25

*Note:* The *p*‐values are reported in the Results text and in the supplemental information.

Across all RRA‐based methods, the *Digestion* model was the most predictive for quantitative deer diet analysis. The *Composition* models were much less supported (Table 3), but in contrast to raw correlations, plant biomass was a significant predictor of RRA in our statistical models (Table S4). This was also true for RGC when using metagenomic sequencing, but the *Composition* model received the strongest support for hybridization capture, with little evidence that accounting for plant digestibility improved model performance. For recreated diets, the *Composition* model was most supported for all molecular methods and both RRA and RGC, with the exception of DNA metabarcoding, for which the *Molecular* model was higher ranked than *Composition* but only by one unit of AIC (Table 3).

**TABLE 3:** Model selection results for DNA metabarcoding, metagenomic sequencing, and hybridization capture. Model support was evaluated using AIC (ΔAIC), while marginal (*R* ^2^m) and conditional (*R* ^2^c) *R* ^2^ values are provided to describe variance explained by fixed effects and by the combined fixed and species‐level random effect, respectively. Across all methods, RRA models for deer diet all supported accounting for digestibility. This was also true for relative genome coverage when using metagenomic sequencing, but hybridization capture models supported plant consumption only, suggesting that this approach overcame biases induced by digestibility. Recreated diet models supported accounting for plant consumption or composition across all models with the exception of DNA metabarcoding, for which including molecular covariates was supported in the best model.

Analysis	Model	AIC	dAIC	*R* ^2^m	*R* ^2^c
*Deer Diet RRA*					
DNA metabarcoding	**Digestion**	**279.75**	**0.00**	**0.49**	**0.78**
	Full	281.00	1.25	0.49	0.79
	Molecular	290.65	10.90	0.26	0.83
	Composition	291.69	11.93	0.30	0.84
Metagenomic sequencing	**Digestion**	**315.96**	**0.00**	**0.49**	**0.83**
	Full	318.18	2.22	0.46	0.85
	Molecular	327.45	11.49	0.32	0.88
	Composition	328.43	12.46	0.33	0.88
Hybridization capture	**Digestion**	**302.02**	**0.00**	**0.44**	**0.80**
	Full	303.67	1.65	0.45	0.80
	Molecular	311.47	9.45	0.30	0.83
	Composition	312.77	10.75	0.28	0.84
*Deer Diet RGC*					
Metagenomic sequencing	**Digestion**	**291.17**	**0.00**	**0.37**	**0.74**
	Full	294.82	3.66	0.35	0.77
	Composition	299.37	8.20	0.22	0.79
	Molecular	301.39	10.22	0.21	0.80
Hybridization capture	**Composition**	**179.08**	**0.00**	**0.45**	**0.78**
	Digestion	183.72	4.64	0.42	0.78
	Molecular	187.43	8.35	0.42	0.78
	Full	190.13	11.04	0.47	0.79
*Plant RRA*					
DNA metabarcoding	**Molecular**	**267.70**	**0.00**	**0.64**	**0.82**
	Composition	268.85	1.15	0.52	0.78
	Full	268.86	1.16	0.52	0.78
	Digestion	269.11	1.41	0.63	0.84
Metagenomic sequencing	**Composition**	**282.64**	**0.00**	**0.59**	**0.85**
	Digestion	285.41	2.77	0.59	0.86
	Molecular	288.08	5.44	0.55	0.86
	Full	289.70	7.07	0.55	0.87
Hybridization capture	**Composition**	**293.89**	**0.00**	**0.49**	**0.85**
	Digestion	296.13	2.24	0.46	0.85
	Molecular	297.78	3.89	0.44	0.85
	Full	298.65	4.76	0.45	0.87
*Plant RGC*					
Metagenomic sequencing	**Composition**	**192.17**	**0.00**	**0.06**	**0.92**
	Digestion	192.80	0.62	0.35	0.95
	Full	197.38	5.20	0.33	0.96
	Molecular	197.65	5.48	0.06	0.94
Hybridization capture	**Composition**	**168.97**	**0.00**	**0.30**	**0.89**
	Digestion	171.01	2.05	0.54	0.92
	Molecular	176.55	7.59	0.29	0.90
	Full	178.35	9.38	0.50	0.93

*Note:* The *p*‐values are reported in the Results text and in the supplemental information.

**FIGURE 4:**
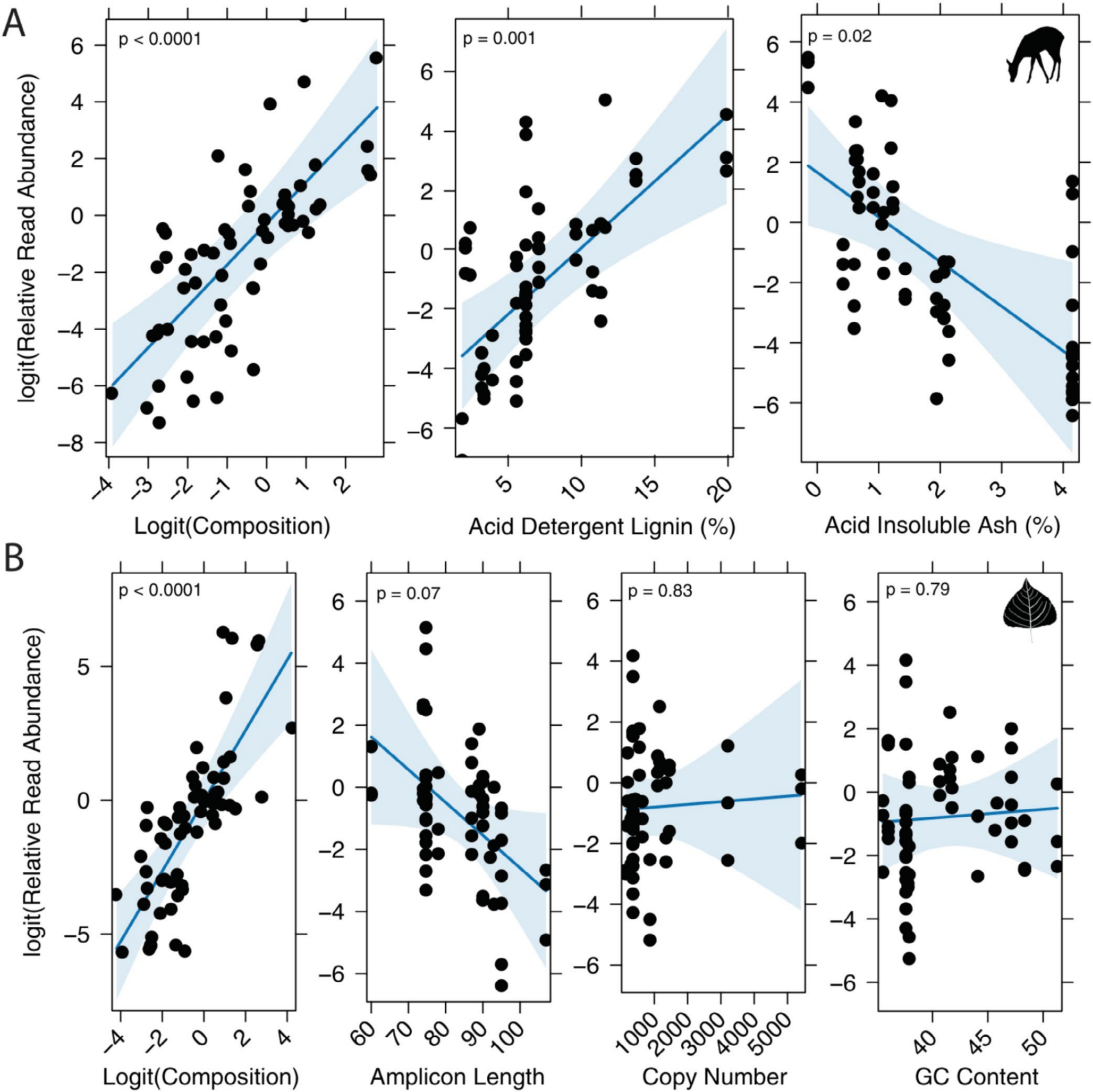
DNA metabarcoding effect sizes for the best supported model for (A) deer diets (consumed by captive mule deer ( *Odocoileus hemionus* ) and white‐tailed deer ( *Odocoileus virginianus* ) in controlled feeding trials), and (B) recreated diets with partial residuals overlaid. Relative read abundance of deer diets increased with higher lignin (ADL) and declined with acid‐insoluble ash (AIA). Accounting for these digestibility covariates revealed a strong relationship between plant consumption and relative read abundance. Relative read abundance of recreated diets was closely related to plant composition and declined with amplicon length but was invariant to GC content and chloroplast copy number.

When accounting for the proportional biomass consumed in deer diets, DNA metabarcoding RRA increased sharply with higher ADL, which is negatively correlated with digestibility (estimate = 1.90, *p* = 0.001, Figure 4A), and declined with AIA (estimate = −2.16, *p* = 0.02, Figure 4A; full model coefficients in Table S4). RRA was strongly predicted by proportional biomass consumed (estimate = 1.46, *p* = 10^−5^), despite weak raw correlations (*R* ^2^ = 0.009). After accounting for bias, the *Digestion* model's fixed effects explained 49% of the variation in diet estimates, and the random effect for species increased the total explained variance to 78%, indicating remaining species‐specific variation. The commercial pellets were considered as a potential source of bias given their processed nature, but the species‐level random effect showed that deviations for pellets were comparable to or smaller than deviations for the different plant species (Figure S3), suggesting that the pellets alone were not driving the results.

For recreated diets, DNA metabarcoding was the only approach for which the *Molecular* model was most predictive (Table 3) (*R* ^2^m = 0.64, *R* ^2^c = 0.82), which was driven by lower RRA for species with longer amplicons (estimate = −1.14, *p* = 0.07) but not GC content (estimate = 0.13, *p* = 0.79) or chloroplast copy number (estimate = 0.11, *p* = 0.83) (Figure 4B and Table S4).

**FIGURE 5:**
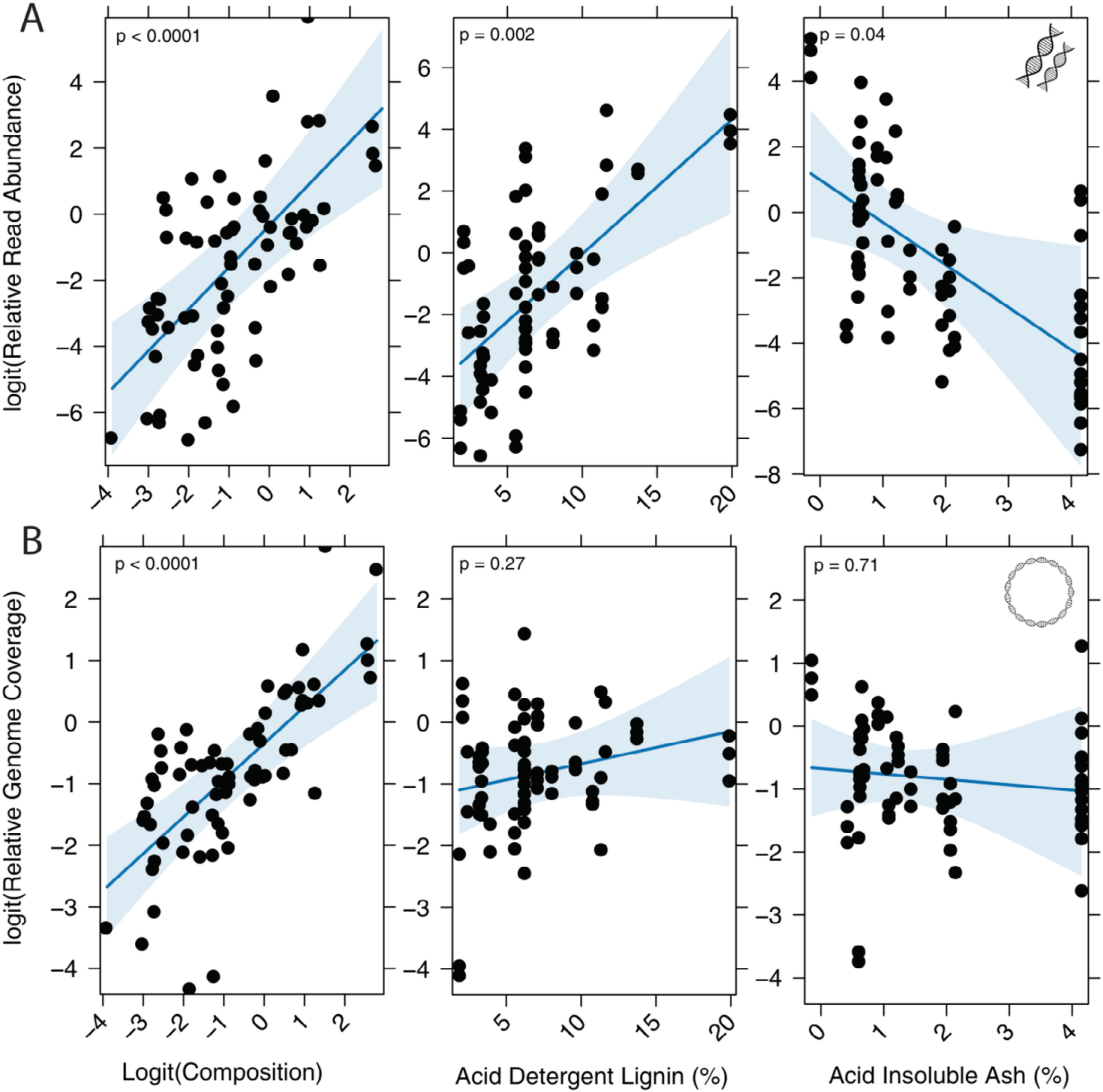
Hybridization capture results for the *Digestion* model using (A) RRA and (B) RGC. RRA results were strongly influenced by digestibility as with DNA metabarcoding and metagenomic sequencing, but RGC overcame these digestibility‐related biases.

Deer diet analysis using metagenomic sequencing and hybridization capture based on RRA was similarly influenced by ADL (Metagenomics: estimate = 2.21, *p* = 0.002, Hybridization: estimate = 1.81, *p* = 0.002) and AIA (Metagenomics: estimate = −2.48, *p* = 0.02, Hybridization: estimate = −1.87, *p* = 0.04; full model coefficients in Table S4). Both methods revealed a strong relationship with proportional plant consumption (Metagenomics: estimate = 1.64, *p* = 10^−6^, Hybridization: estimate = 1.27, *p* = 10^−5^). Hybridization results are shown in Figure 5A, metagenomics in Table S4. These digestibility metrics retained a similar influence on RGC for metagenomic sequencing (Table S5 and Figure S2) but not for hybridization capture (ADL: estimate = 0.22, *p* = 0.27, AIA: estimate = −0.12, *p* = 0.71; Figure 5B). All other effect sizes are presented in Figure S2.

The presence of closely related taxa in the reference library did not significantly bias relative abundance estimates in most models. For scat samples, no effects were detected for hybridization capture (RRA: estimate = −0.72, *p* = 0.3; RGC: estimate = −0.05, *p* = 0.86) or metagenomic sequencing (RRA: estimate = −0.14, *p* = 0.86; RGC: estimate = 0.2, *p* = 0.75). Likewise, metagenomic sequencing of recreated diet samples showed no effect (RRA: estimate = −0.50, *p* = 0.3; RGC: estimate = −0.50, *p* = 0.11). However, there was a marginally significant effect for hybridization capture using RRA (estimate = −1.03, *p* = 0.06) with recreated diet samples, but not using RGC (estimate = −0.27, *p* = 0.22).

## 4 Discussion

Despite widespread use of DNA metabarcoding and RRA for herbivore diet analysis (e.g., Gill et al. 2023; ter Schure et al. 2021; Mas‐Carrió et al. 2024), our results indicate that RRA provides limited within‐sample quantitative information without correction. Across all three molecular methods, RRA was uncorrelated with proportional plant consumption by deer (Figure 2), suggesting that herbivore diet analysis using uncorrected RRA is inaccurate. However, RRA was strongly correlated with plant composition in the recreated diet samples (Figure 2), indicating that the source of bias is not primarily molecular properties such as GC content, amplicon length, or chloroplast copy number. Our statistical models strongly supported differential plant digestibility, and not molecular biases, as the main source of bias in the RRA of deer diets, and accounting for digestibility through ADL (lignin and cutin) and AIA (acid‐insoluble ash) substantially improved the relationship between plant consumption and RRA. Although molecular biases were weak relative to digestibility, metabarcoding of recreated diet samples suggested that RRA declined somewhat with amplicon length, presumably due to reduced amplification efficiency or potentially overrepresentation of short reads on Illumina flow cells. We found no evidence that GC content or chloroplast copy number influenced RRA.

Importantly, these patterns suggest that RRA is not simply uninformative in scat samples, but that its signal is masked by systematic biases rather than random noise. Our mixed‐effects models indicate that digestibility explains a substantial portion of this bias, and accounting for ADL and AIA can improve inference from molecular diet analysis. However, substantial residual variation remains attributable to species‐level effects, as indicated by the strong contribution of random intercepts in the digestion models, suggesting that correction improves accuracy substantially but does not fully explain species‐level variation.

**FIGURE 6:**
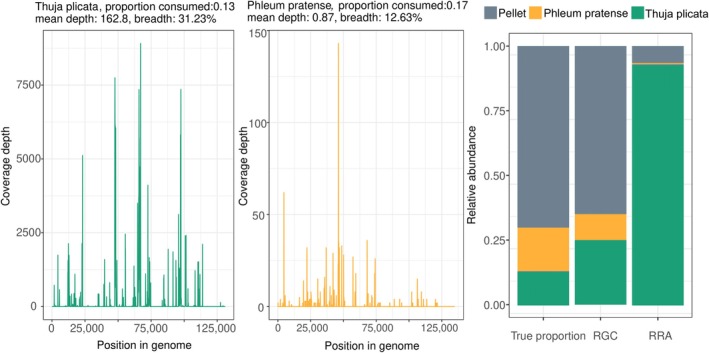
Example of per base depth versus breadth of coverage for *Thuja plicata* and *Phleum pratense* using hybridization capture of a single deer scat sample (S63F) and the corresponding relative diet composition estimates using three metrics: True proportion consumed, Relative Genome Coverage (RGC), Relative Read Abundance (RRA). Note that the y‐axes are scaled differently for each coverage plot. All other samples are shown in Figure S1.

Given that digestibility is the dominant source of bias in RRA, correcting for it has promise to improve inference from molecular diet analysis. However, when such corrections are not feasible because fiber constituents of the relevant plants are unknown, hybridization capture paired with RGC may provide a reliable alternative method for diet quantification. This approach achieved the highest correlation with biomass consumed and the lowest mean absolute error without correcting for digestion. The improved performance of RGC likely reflects its ability to mitigate biases arising from differential DNA survival during digestion by relying on breadth rather than depth of coverage. Species that are less digestible contribute disproportionately more DNA molecules to fecal samples, inflating their representation in read counts. In contrast to RRA, which generally increases with the number of recovered DNA molecules, genome coverage is a bounded metric (0–1) that follows a saturating relationship with sequencing depth. As genome coverage approaches saturation, additional reads contribute relatively little to RGC, compressing differences among species and potentially reducing bias in relative abundance estimates (Figure 6). In addition, although hybridization capture enriches dietary DNA across many loci, it can still result in uneven coverage due to differences in probe efficiency and DNA quality (Hawkins et al. 2016). RGC reduces the effects of these biases by focusing on how broadly the genome is covered, rather than depth at a few overrepresented regions, making it more robust to stochastic and technical variation in locus recovery arising from DNA degradation and capture efficiency in fecal samples compared to RRA. By focusing on genome‐wide coverage, RGC may also improve the recovery of underrepresented taxa that still had broad genomic representation but relatively few total reads (Figure 6). It was also the only method that showed resilience against digestion‐related biases that inflated fibrous, indigestible species (e.g., *Salix* stems, *Thuja* needles) in the other methods. In contrast, RGC did not improve diet quantification from metagenomic sequencing of scat samples. Unlike hybridization capture, metagenomics sequences all DNA without enrichment, and chloroplast DNA therefore represent only a small fraction of the total reads. This makes recovery of chloroplast sequences more stochastic, particularly for rare or highly digestible species. For example, *Brassica* was not detected in any scat metagenomics samples. Consequently, even though metagenomics avoids PCR bias, low and uneven chloroplast recovery and insufficient sequencing depth (Woo et al. 2023), likely limited the accuracy of both RRA and RGC.

Accounting for species‐specific biases (e.g., consistent underestimation of *Brassica* or overestimation of *Thuja*) would help improve herbivore diet analysis. These correction factors can be derived from linear models such as that used here by solving for proportion consumed as a function of RRA and digestibility metrics. For example, the proportion of species *i* within a sample can be back calculated as a function of indigestible plant fiber components (ADL) and minerals (AIA) as,

$$P_{i}=\frac{1}{1+\exp\left(-\left(\frac{\text{logit}\left({\mathrm{RRA}}_{i}\right)-\beta_{0}-\beta_{2}{\mathrm{ADL}}_{i}-\beta_{3}{\mathrm{AIA}}_{i}}{\beta_{1}}\right)\right)}$$

and then renormalized among species. Using our model with standardized covariates (coefficients provided in Table S4 for RRA and Table S5 for RGC) can facilitate correction when digestibility covariates are unknown but can be approximated into categories. Estimates of proportion consumed can be adjusted for plants with average digestibility using ADL as a proxy (ADL = 0; e.g., rose leaves and petioles), (±1 SD) for plants with well above (strawberry leaves) or below (sagebrush leaves) average digestibility, or (±2 SD) for extremely undigestible (conifer needles) or digestible (fresh kale) plants. For example, consider a sample with two plants, one with RRA_1_ = 0.3 and the second with RRA_2_ = 0.7. If plant 1 has low digestibility due to high lignin (ADL= 1) while plant 2 has high digestibility due to low lignin (ADL= −1), and holding AIA at the mean (AIA= 0), the predicted dietary proportions of species 1 and 2 become *P*_1_ = 0.15 and *P*_2_ = 0.85 after renormalization. Alternatively, the coefficient values for the equivalent model with unstandardized covariates (Table S6 for RRA; Table S7 for RGC) enables more precise estimation of proportion consumed when digestibility covariates are known.

In conclusion, we found that the widely used RRA metric produced biased inference on animal diets primarily due to differential digestibility rather than molecular effects. Correcting for digestibility using plant fiber metrics substantially increases quantitative accuracy and should be prioritized when possible. When such corrections are not feasible, hybridization capture paired with RGC may be a promising alternative for improving accuracy and taxonomic resolution without explicit bias correction. However, from a practical standpoint, method choice should be driven by study objectives and resource availability. While hybridization capture may be superior in quantitative accuracy and taxonomic resolution, it remains costlier and more labor‐intensive, making it most useful for studies demanding precise diet reconstruction from degraded samples. Further research is needed to assess the generality of this approach across different probe sets, sequencing depth, lab protocols, and species assemblages. DNA metabarcoding, though less quantitatively and taxonomically robust, offers scalability and can achieve sufficient accuracy when paired with careful primer selection (particularly avoiding primer‐template mismatches near the 3′ end), large sample sizes (because frequency of occurrence adds quantitative signal), and controlling for differential digestibility. Building reference databases of species‐specific digestibility covariates could support correction factors that extend the quantitative utility of metabarcoding to a broader range of ecological studies.

## Author Contributions

C.E.E., T.L. and L.S. conceived the research. L.S. led deer experiments, plant sampling and nutritional analysis. C.E.E. performed laboratory work and bioinformatic analysis. T.L. performed DNA metabarcoding bioinformatic analysis. D.A.C. provided funding for the research. C.E.E. wrote the first version of the paper, and all authors provided edits and comments.

## Funding

This work was supported by USDA National Institute of Food and Agriculture, McIntire‐Stennis Project, WNP00848. Federal Aid in Wildlife Restoration, F23AF03162. National Science Foundation, 2317537.

## Conflicts of Interest

The authors declare no conflicts of interest.

## Supporting information


**Figure S1:** Composition of feeding trial diets and corresponding estimates of relative abundance for each deer sample (*n* = 25). One scat sample failed library preparation and is therefore not included. Stacked bar plots show the proportion of each plant species in individual feeding trial samples (x‐axis), with colours representing plant taxa. Panels are separated by method: (A) DNA metabarcoding*, (B) Metagenomic sequencing, (C) Hybridization capture. *The three Rosaceae species (*F. ananassa*, *R. pacificus* , and *R. canina* ) in diet 9 could not be distinguished with DNA metabarcoding due to identical trnL sequences and were therefore pooled.
**Figure S2:** Effect sizes (standardized regression coefficients ±95% CI) from linear mixed‐effects models predicting relative read abundance (A) or relative genomic coverage (B) across molecular methods and sample types. Models include predictors related to plant digestibility (acid detergent lignin (ADL, %), and acid insoluble ash (AIA, %)), molecular characteristics (amplicon/genome length, GC content, chloroplast copy number), and proportional plant biomass consumed (deer diets) or used in diet reconstruction (recreated diets).
**Figure S3:** Random intercepts from the Digestion model for each plant species across methods and metrics in deer scat samples. The top row shows RRA (relative read abundance) and the bottom row shows RGC (relative genome coverage). Each point represents a species' deviation from the average predicted diet proportion; species with positive values are overrepresented, and those with negative values are underrepresented.
**Table S1:** Species and Genbank accession IDs used in the chloroplast genome reference library for Kraken2 and Minimap2 analysis. *indicates the use of a congener since the chloroplast genome was not available for the feeding trial species at the time of analysis.
**Table S2:** Kraken2 confidence scores and detection thresholds based on fraction of reads. ‘*N* species’ = number of species‐sample combinations detected prior to applying filtering threshold, ‘frac filter’ = Filtering threshold based on fraction of reads assigned, “FP num” = number of false positives, “FN num” = number of false negatives, “*N* species final” = Number of species‐sample combinations after filtering.
**Table S3:** Read mapping method with detection thresholds based on proportion genome coverage. ‘*N* species’ = number of species‐sample combinations detected prior to applying filtering threshold, ‘prop filter’ = Filtering threshold based on proportion genome coverage, “FP num” = number of false positives, “FN num” = number of false negatives, “*N* species final” = Number of species‐sample combinations after filtering.
**Table S4:** Fixed‐effect coefficients from linear mixed‐effects models using logit‐transformed Relative Read Abundance (RRA) as the response variable. Models were fitted separately for each molecular method (DNA metabarcoding, metagenomic sequencing, and hybridization capture) and sample type (deer scats and recreated diets). Each model included a random intercept for plant species and pellet type to account for repeated measures. The Composition model included only the logit‐transformed proportion of plant biomass consumed (for scat samples) or used to construct the recreated diet sample. The Digestion model extended this by including percent acid detergent lignin (ADL), and acid insoluble ash (AIA). The Molecular model included covariates representing potential molecular biases: GC content (gc) of the trnL amplicon or full chloroplast genome, amplicon or chloroplast genome length (length), and estimated chloroplast copy number (copies). The Full model included all digestibility and molecular covariates. All continuous predictors were standardized (mean = 0, SD = 1) to allow comparison of effect sizes across covariates.
**Table S5:** Fixed‐effect coefficients from linear mixed‐effects models using logit‐transformed Relative Genome Coverage (RGC) as the response variable. Models were fit separately by molecular method and sample type, with plant species and pellet type as a random intercept. Covariate descriptions and model structures (Consumption, Digestion, Molecular, and Full) are provided in the Table S4 caption. All continuous covariates were standardized (mean = 0, SD = 1).
**Table S6:** Fixed‐effect coefficients from linear mixed‐effects models using logit‐transformed Relative Read Abundance (RRA) as the response variable, with covariates in their original (unstandardized) units. Model structure and covariate definitions follow those described in Table S4.
**Table S7:** Fixed‐effect coefficients from linear mixed‐effects models using logit‐transformed Relative Genome Coverage (RGC) as the response variable, with covariates in their original (unstandardized) units. Model structure and covariate definitions follow those described in Table S4.

## Data Availability

Data and associated code have been deposited in Dryad at: https://doi.org/10.5061/dryad.np5hqc06j. *Benefits Sharing Statement*: The benefits of this research accrue from the sharing of our data and results in public databases, as described above.
