# Supplementary material for: Comparing Accuracy and Biases of DNA Metabarcoding, Hybridization Capture, and Metagenomic Sequencing for Quantifying Herbivore Diets
Source: Mol Ecol Resour. 2026 Jul 16;26(5):e70175. doi: 10.1111/1755-0998.70175 (PMC13376231; doi:10.1111/1755-0998.70175)
Supplement: Supplementary file 1 — Figure S1: Composition of feeding trial diets and corresponding estimates of relative abundance for each deer sample (n = 25). One scat sample failed library preparation and is therefore not included. Stacked bar plots show the proportion of each plant species in individual feeding trial samples (x‐axis), with colours representing plant taxa. Panels are separated by method: (A) DNA metabarcoding*, (B) Metagenomic sequencing, (C) Hybridization capture. *The three Rosaceae species (F. ananassa, R. pacificus , and R. canina ) in diet 9 could not be distinguished with DNA metabarcoding due to identical trnL sequences and were therefore pooled. Figure S2: Effect sizes (standardized regression coefficients ±95% CI) from linear mixed‐effects models predicting relative read abundance (A) or relative genomic coverage (B) across molecular methods and sample types. Models include predictors related to plant digestibility (acid detergent lignin (ADL, %), and acid insoluble ash (AIA, %)), molecular characteristics (amplicon/genome length, GC content, chloroplast copy number), and proportional plant biomass consumed (deer diets) or used in diet reconstruction (recreated diets). Figure S3: Random intercepts from the Digestion model for each plant species across methods and metrics in deer scat samples. The top row shows RRA (relative read abundance) and the bottom row shows RGC (relative genome coverage). Each point represents a species' deviation from the average predicted diet proportion; species with positive values are overrepresented, and those with negative values are underrepresented. Table S1: Species and Genbank accession IDs used in the chloroplast genome reference library for Kraken2 and Minimap2 analysis. *indicates the use of a congener since the chloroplast genome was not available for the feeding trial species at the time of analysis. Table S2: Kraken2 confidence scores and detection thresholds based on fraction of reads. ‘N species’ = number of species‐sample combi [file MEN-26-e70175-s001.docx]

**Supplemental Information**

**Comparing accuracy and biases of DNA metabarcoding, hybridization capture, and metagenomic sequencing for quantifying herbivore diets**

Charlotte E. Eriksson^1^, Lisa Shipley^2^, Darren A. Clark^,3^, Taal Levi^1^

Table S1. Species and Genbank accession IDs used in the chloroplast genome reference library for Kraken2 and Minimap2 analysis. * indicates the use of a congener since the chloroplast genome was not available for the feeding trial species at the time of analysis.

| **Accession ID** | **Scientific name** |
| --- | --- |
| MT906790.1 | *Acer platanoides* |
| NC_067048.1 | *Alopecurus pratensis* |
| NC_042673.1 | *Arrhenatherum elatius* |
| NC_070204.1 | *Artemisia santonicum** |
| MG687313.1 | *Avena sativa* |
| EF534108.1 | *Beta vulgaris* |
| NC_041167.1 | *Brassica oleracea* |
| MN481508.1 | *Chamaenerion angustifolium* |
| OP764691.1 | *Fragaria x ananassa* |
| NC_007942.1 | *Glycine max* |
| NC_056985.1 | *Hordeum vulgare* |
| NC_036356.1 | *Linum usitatissimum* |
| NC_061549.1 | *Malus domestica* |
| OP514753.1 | *Medicago sativa* |
| NC_031333.1 | *Oryza sativa* |
| NC_067044.1 | *Phleum pratense* |
| NC_058279.1 | *Populus tremuloides* |
| NC_039989.1 | *Rosa multiflora* |
| NC_064142.1 | *Rubus pacificus* |
| NC_035224.1 | *Saccharum officinarum* |
| NC_026462.1 | *Salix suchowensis** |
| NC_045061.1 | *Sambucus nigra* |
| NC_042175.1 | *Thuja plicata* |
| NC_001666.2 | *Zea mays* |

Table S2. Kraken2 confidence scores and detection thresholds based on fraction of reads. ‘N species’ = number of species-sample combinations detected prior to applying filtering threshold, ‘frac filter’ = Filtering threshold based on fraction of reads assigned, “FP num” = number of false positives, “FN num” = number of false negatives, “N species final” = Number of species-sample combinations after filtering.

| **Method** | **Sample type** | **Confidence** | **N species** | **N samples** | **frac filter** | **FP num** | **FN num** | **N species final** |
| --- | --- | --- | --- | --- | --- | --- | --- | --- |
| Metagenomic seq | Recreated | 0.05 | 44 | 26 | 0.01 | 0 | 4 | 40 |
| Metagenomic seq | Recreated | 0.05 | 44 | 26 | 0.001 | 24 | 2 | 42 |
| Metagenomic seq | Recreated | 0.1 | 44 | 26 | 0.01 | 0 | 4 | 40 |
| Metagenomic seq | Recreated | 0.1 | 44 | 26 | 0.001 | 13 | 2 | 42 |
| Metagenomic seq | Recreated | 0.2 | 44 | 26 | 0.01 | 0 | 7 | 37 |
| Metagenomic seq | Recreated | 0.2 | 44 | 26 | 0.001 | 0 | 2 | 42 |
| Metagenomic seq | Recreated | 0.5 | 44 | 26 | 0.01 | 0 | 7 | 37 |
| Metagenomic seq | Recreated | 0.5 | 44 | 26 | 0.001 | 0 | 2 | 42 |
| Hybridization cap | Recreated | 0.05 | 46 | 26 | 0.01 | 1 | 6 | 40 |
| Hybridization cap | Recreated | 0.05 | 46 | 26 | 0.001 | 83 | 3 | 43 |
| Hybridization cap | Recreated | 0.1 | 46 | 26 | 0.01 | 1 | 6 | 40 |
| Hybridization cap | Recreated | 0.1 | 46 | 26 | 0.001 | 50 | 3 | 43 |
| Hybridization cap | Recreated | 0.2 | 46 | 26 | 0.01 | 1 | 6 | 40 |
| Hybridization cap | Recreated | 0.2 | 46 | 26 | 0.001 | 25 | 3 | 43 |
| Hybridization cap | Recreated | 0.5 | 45 | 26 | 0.01 | 1 | 6 | 39 |
| Hybridization cap | Recreated | 0.5 | 45 | 26 | 0.001 | 10 | 3 | 42 |

Table S3. Read mapping method with detection thresholds based on proportion genome coverage. ‘N species’ = number of species-sample combinations detected prior to applying filtering threshold, ‘prop filter’ = Filtering threshold based on proportion genome coverage, “FP num” = number of false positives, “FN num” = number of false negatives, “N species final” = Number of species-sample combinations after filtering.

| **Method** | **Sample type** | **N species** | **N samples** | **prop filter** | **FP num** | **FN num** | **N species final** |
| --- | --- | --- | --- | --- | --- | --- | --- |
| Metagenomic seq | Recreated | 44 | 26 | 0.01 | 100 | 0 | 44 |
| Metagenomic seq | Recreated | 44 | 26 | 0.05 | 25 | 3 | 41 |
| Metagenomic seq | Recreated | 44 | 26 | 0.1 | 11 | 4 | 40 |
| Hybridization cap | Recreated | 46 | 26 | 0.01 | 129 | 0 | 46 |
| Hybridization cap | Recreated | 46 | 26 | 0.05 | 15 | 3 | 43 |
| Hybridization cap | Recreated | 46 | 26 | 0.1 | 7 | 7 | 39 |


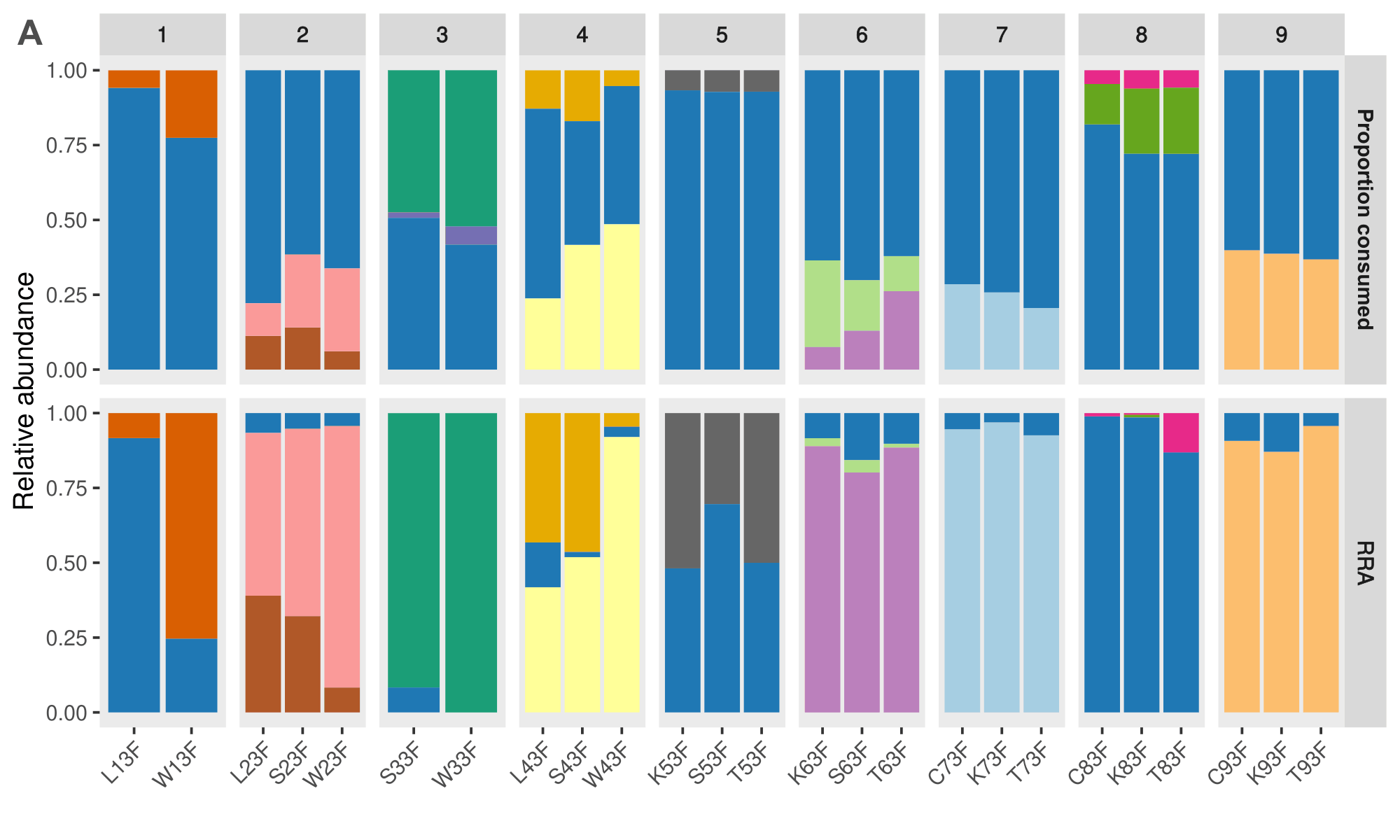

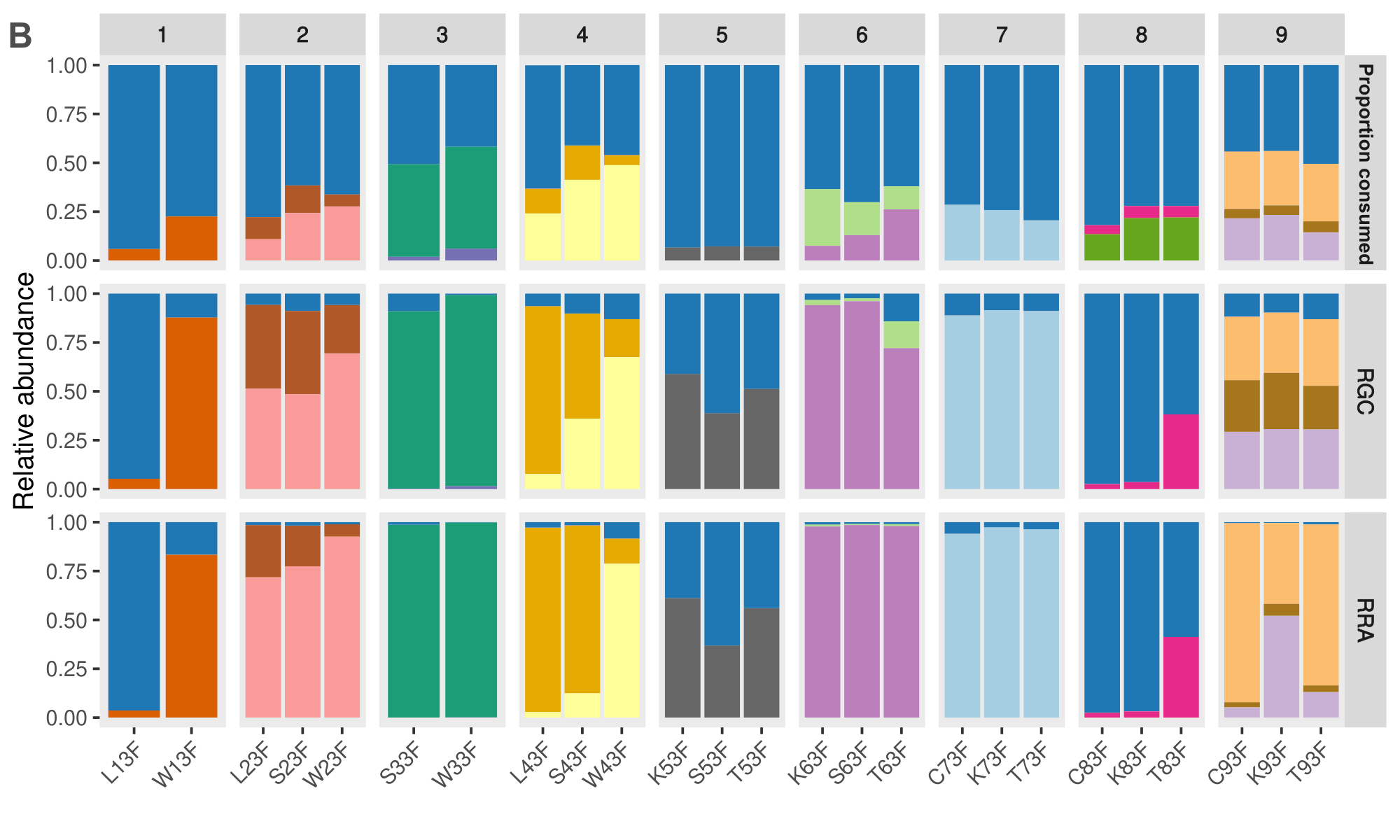

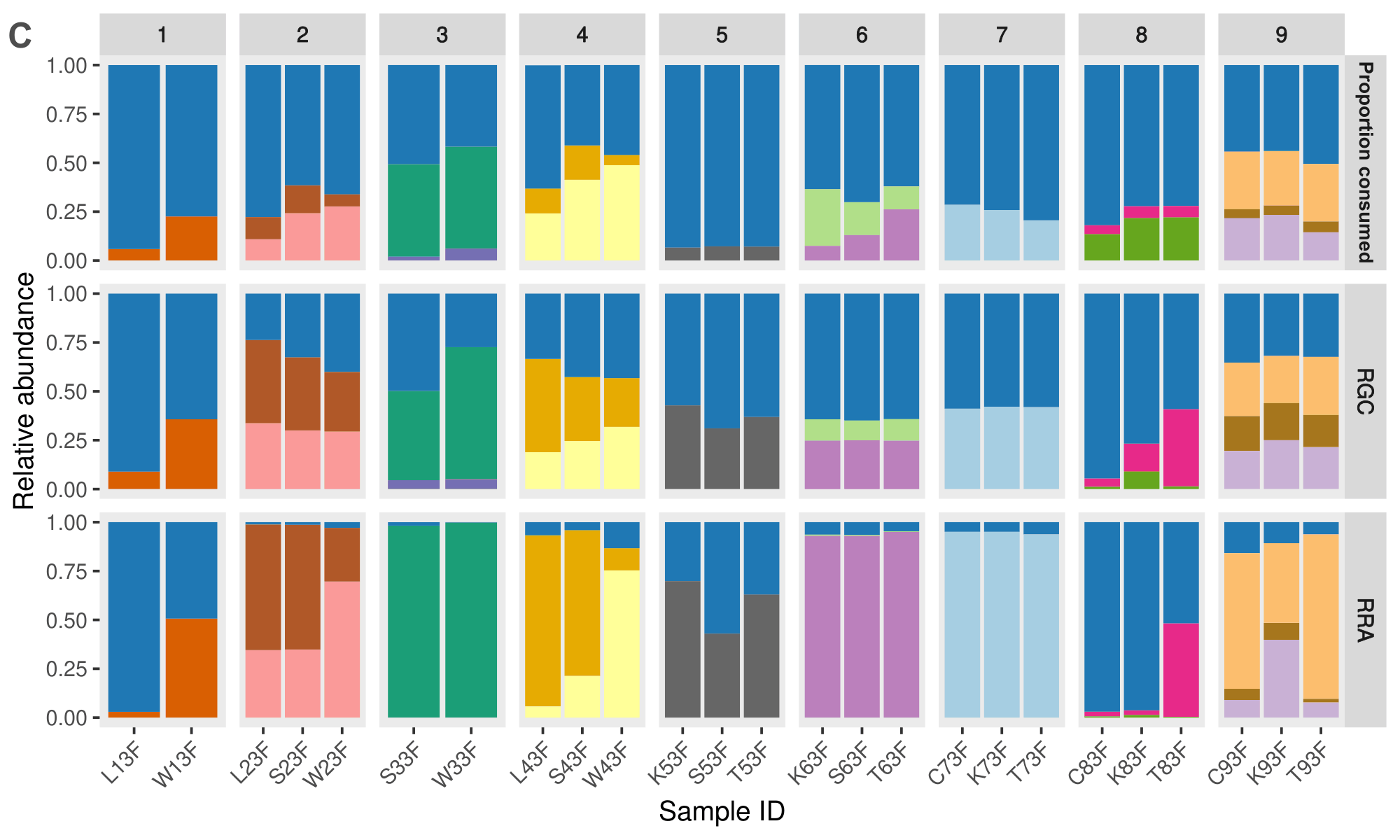


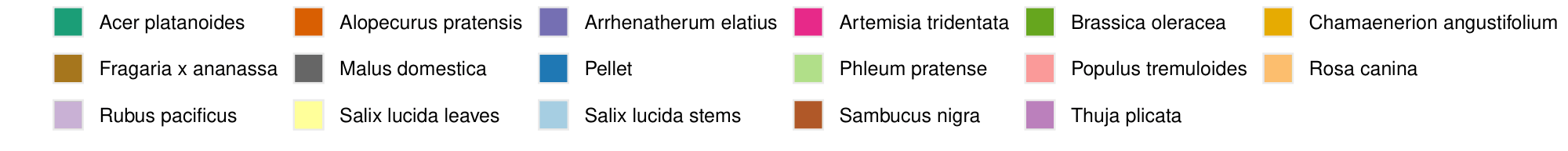


Figure S1. Composition of feeding trial diets and corresponding estimates of relative abundance for each deer sample (n = 25). One scat sample failed library preparation and is therefore not included. Stacked bar plots show the proportion of each plant species in individual feeding trial samples (x-axis), with colors representing plant taxa. Panels are separated by method: A) DNA metabarcoding*, B) Metagenomic sequencing, C) Hybridization capture. *The three Rosaceae species (*F. ananassa*, *R. pacificus*, and *R. canina*) in diet 9 could not be distinguished with DNA metabarcoding due to identical trnL sequences and were therefore pooled.


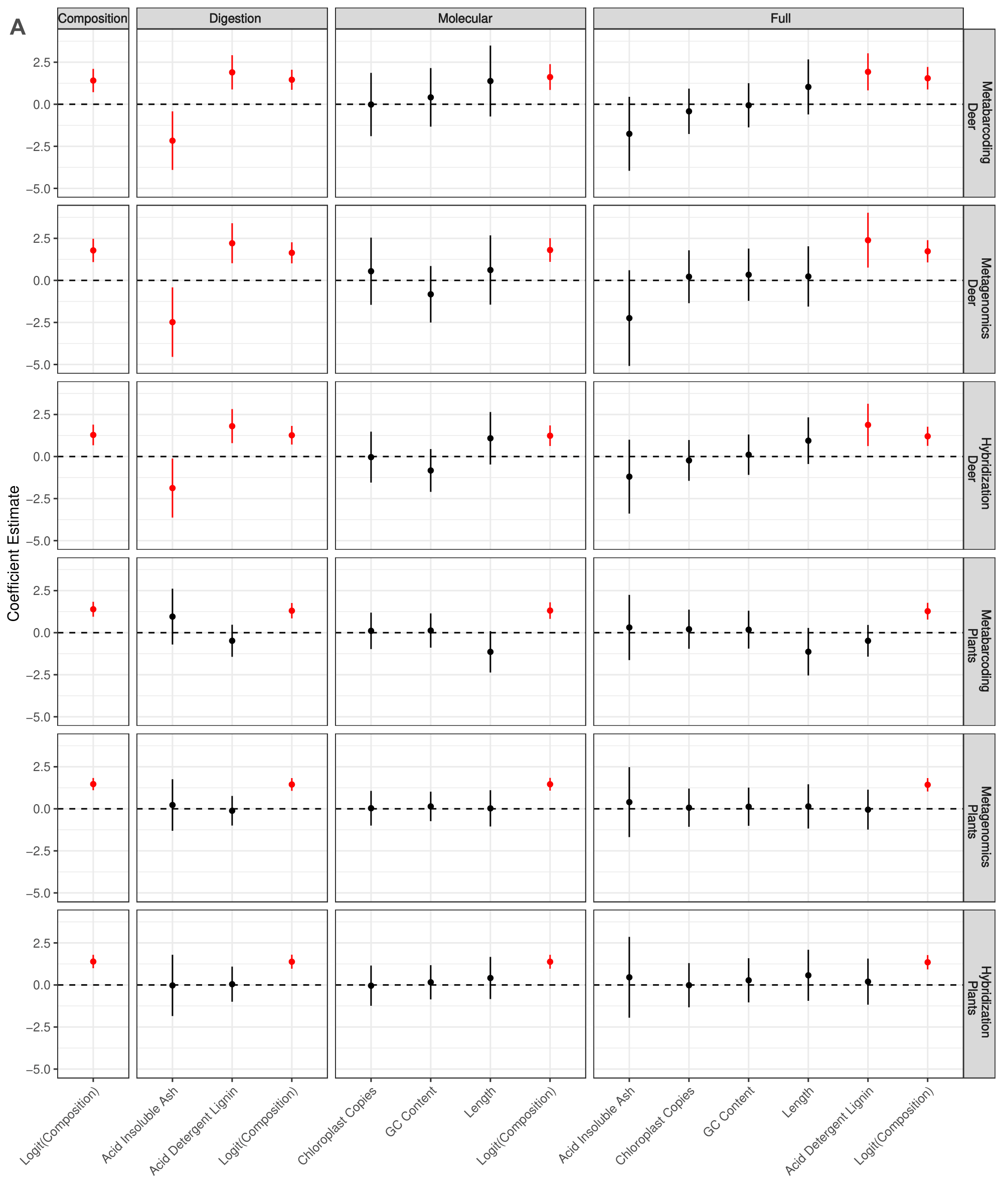


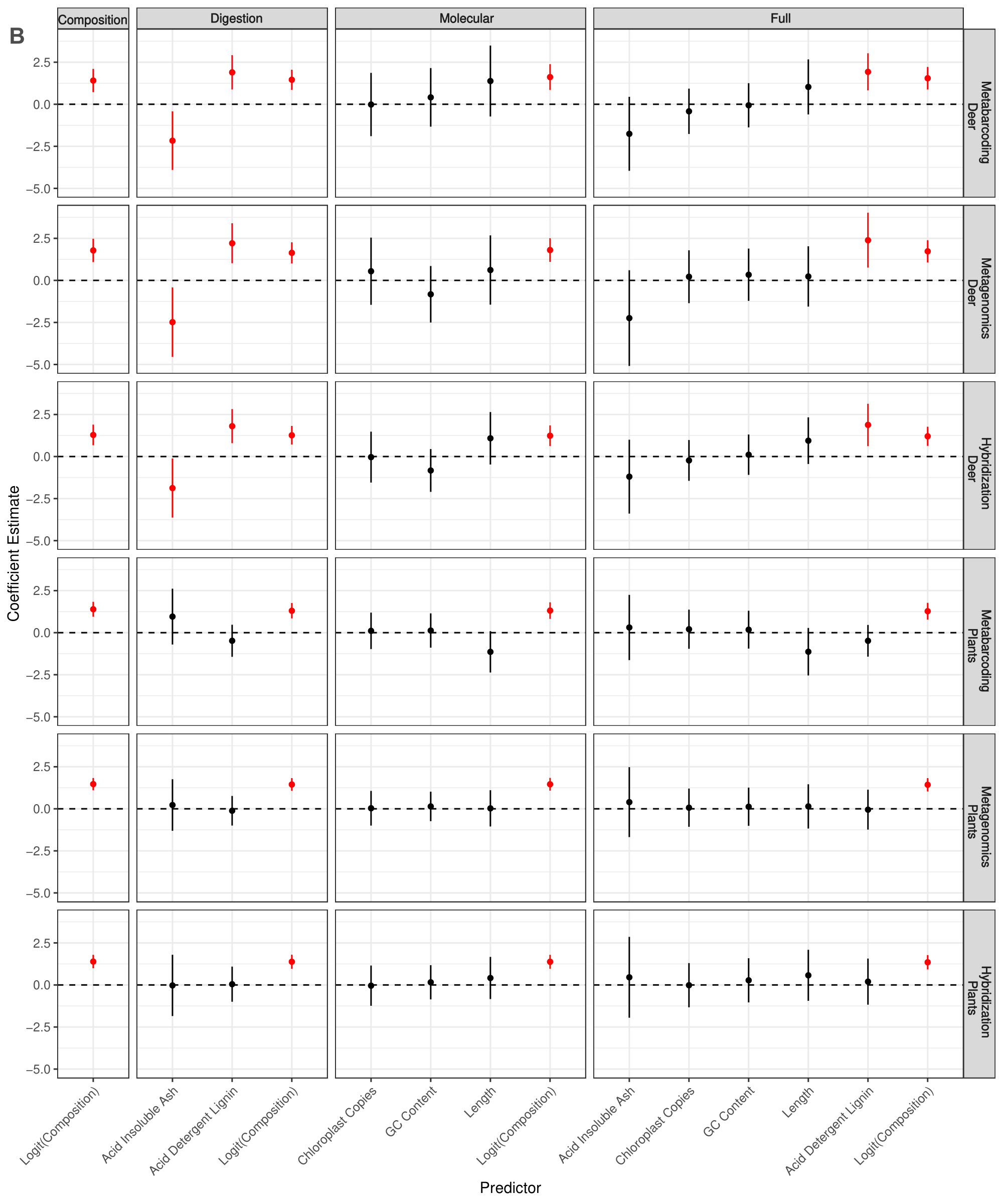
Figure S2. Effect sizes (standardized regression coefficients ± 95% CI) from linear mixed-effects models predicting relative read abundance (A) or relative genomic coverage (B) across molecular methods and sample types. Models include predictors related to plant digestibility (acid detergent lignin (ADL, %), and acid insoluble ash (AIA, %)), molecular characteristics (amplicon/genome length, GC content, chloroplast copy number), and proportional plant biomass consumed (deer diets) or used in diet reconstruction (recreated diets).


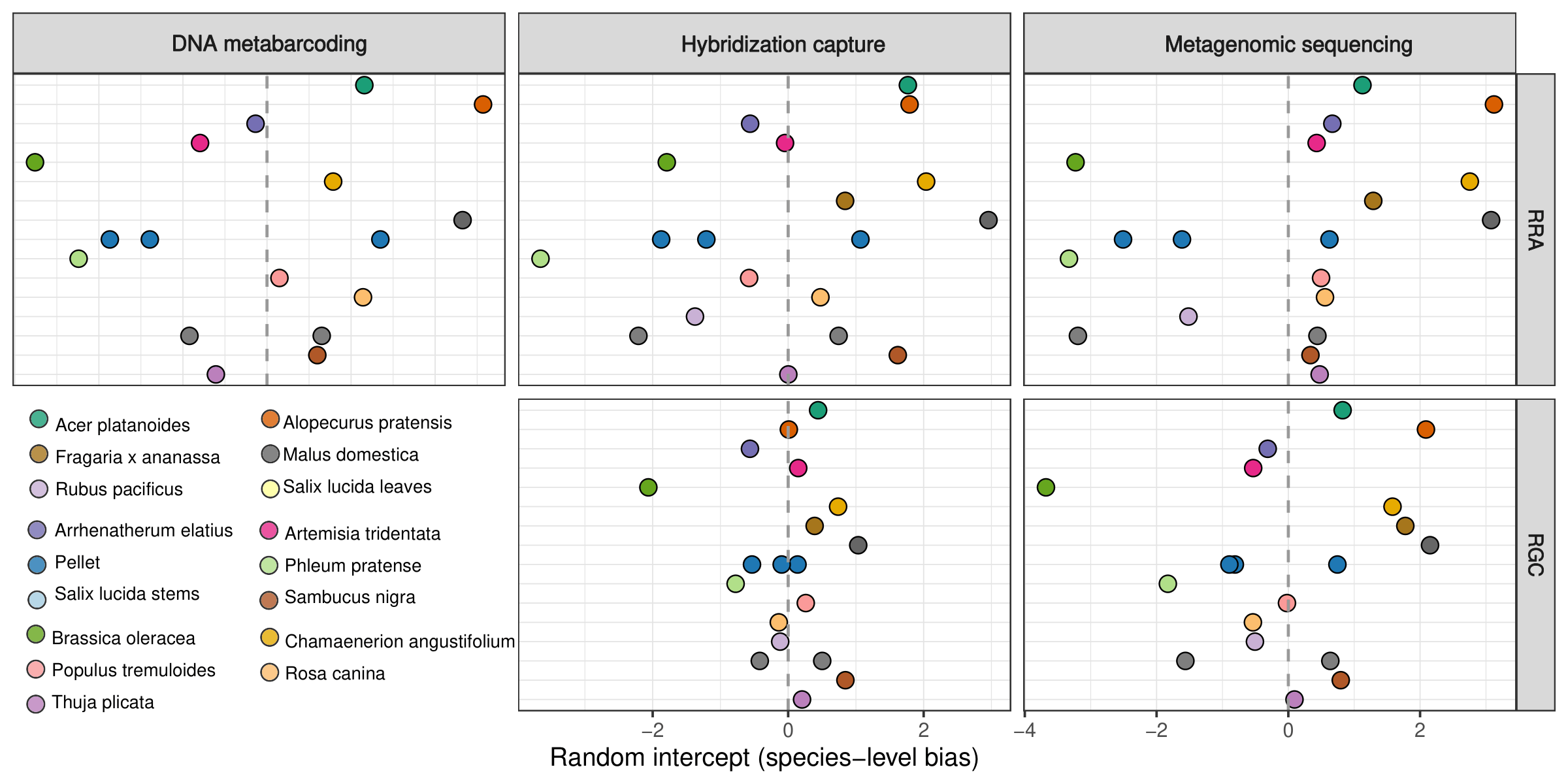


Figure S3. Random intercepts from the *Digestion* model for each plant species across methods and metrics in deer scat samples. The top row shows RRA (relative read abundance) and the bottom row shows RGC (relative genome coverage). Each point represents a species’ deviation from the average predicted diet proportion; species with positive values are overrepresented, and those with negative values are underrepresented.

Table S4. Fixed-effect coefficients from linear mixed-effects models using logit-transformed Relative Read Abundance (RRA) as the response variable. Models were fitted separately for each molecular method (DNA metabarcoding, metagenomic sequencing, and hybridization capture) and sample type (deer scats and recreated diets). Each model included a random intercept for plant species and pellet type to account for repeated measures. The Composition model included only the logit-transformed proportion of plant biomass consumed (for scat samples) or used to construct the recreated diet sample. The Digestion model extended this by including percent acid detergent lignin (ADL), and acid insoluble ash (AIA). The Molecular model included covariates representing potential molecular biases: GC content (gc) of the trnL amplicon or full chloroplast genome, amplicon or chloroplast genome length (length), and estimated chloroplast copy number (copies). The Full model included all digestibility and molecular covariates. All continuous predictors were standardized (mean = 0, SD = 1) to allow comparison of effect sizes across covariates.

| **dataset_name** | **model_name** | **effect** | **term** | **estimate** | **std.error** | **statistic** | **df** | **p.value** |
| --- | --- | --- | --- | --- | --- | --- | --- | --- |
| Metabarcoding_deer | Composition | fixed | Intercept | 0.74 | 0.85 | 0.86 | 18.60 | 0.3988386 |
| Metabarcoding_deer | Composition | fixed | logit(composition) | 1.41 | 0.35 | 4.07 | 51.63 | 0.0001594 |
| Metabarcoding_deer | Digestion | fixed | Intercept | -0.28 | 0.65 | -0.43 | 12.06 | 0.6735934 |
| Metabarcoding_deer | Digestion | fixed | logit(composition) | 1.46 | 0.29 | 4.98 | 33.72 | 0.0000185 |
| Metabarcoding_deer | Digestion | fixed | scale(corr_adl) | 1.90 | 0.47 | 4.02 | 12.99 | 0.0014608 |
| Metabarcoding_deer | Digestion | fixed | scale(corr_aia) | -2.16 | 0.79 | -2.73 | 11.23 | 0.0192192 |
| Metabarcoding_deer | Molecular | fixed | Intercept | 0.48 | 0.90 | 0.53 | 13.90 | 0.6036845 |
| Metabarcoding_deer | Molecular | fixed | logit(composition) | 1.62 | 0.38 | 4.23 | 56.50 | 0.0000864 |
| Metabarcoding_deer | Molecular | fixed | scale(length) | 1.38 | 0.98 | 1.41 | 13.72 | 0.1813018 |
| Metabarcoding_deer | Molecular | fixed | scale(copies) | -0.01 | 0.87 | -0.02 | 12.48 | 0.9866613 |
| Metabarcoding_deer | Molecular | fixed | scale(gc) | 0.41 | 0.82 | 0.50 | 14.63 | 0.6220471 |
| Metabarcoding_deer | Full | fixed | Intercept | -0.23 | 0.68 | -0.33 | 9.76 | 0.7467251 |
| Metabarcoding_deer | Full | fixed | logit(composition) | 1.55 | 0.33 | 4.67 | 36.52 | 0.0000401 |
| Metabarcoding_deer | Full | fixed | scale(corr_adl) | 1.93 | 0.50 | 3.89 | 10.08 | 0.0029439 |
| Metabarcoding_deer | Full | fixed | scale(corr_aia) | -1.76 | 0.97 | -1.82 | 8.66 | 0.1036823 |
| Metabarcoding_deer | Full | fixed | scale(length) | 1.03 | 0.75 | 1.38 | 11.32 | 0.1935062 |
| Metabarcoding_deer | Full | fixed | scale(copies) | -0.42 | 0.61 | -0.69 | 10.11 | 0.5055865 |
| Metabarcoding_deer | Full | fixed | scale(gc) | -0.06 | 0.60 | -0.10 | 12.00 | 0.9220474 |
| Metabarcoding_plants | Composition | fixed | Intercept | -0.19 | 0.54 | -0.36 | 16.37 | 0.7257994 |
| Metabarcoding_plants | Composition | fixed | logit(composition) | 1.39 | 0.22 | 6.33 | 43.75 | 0.0000001 |
| Metabarcoding_plants | Digestion | fixed | Intercept | 0.15 | 0.61 | 0.24 | 11.38 | 0.8112629 |
| Metabarcoding_plants | Digestion | fixed | logit(composition) | 1.30 | 0.23 | 5.71 | 47.10 | 0.0000007 |
| Metabarcoding_plants | Digestion | fixed | scale(corr_adl) | -0.48 | 0.44 | -1.09 | 12.68 | 0.2947276 |
| Metabarcoding_plants | Digestion | fixed | scale(corr_aia) | 0.96 | 0.75 | 1.27 | 10.92 | 0.2307509 |
| Metabarcoding_plants | Molecular | fixed | Intercept | -0.02 | 0.50 | -0.03 | 11.69 | 0.9735098 |
| Metabarcoding_plants | Molecular | fixed | logit(composition) | 1.31 | 0.24 | 5.38 | 51.37 | 0.0000019 |
| Metabarcoding_plants | Molecular | fixed | scale(length) | -1.14 | 0.57 | -1.99 | 14.15 | 0.0664076 |
| Metabarcoding_plants | Molecular | fixed | scale(copies) | 0.11 | 0.50 | 0.22 | 11.87 | 0.8293293 |
| Metabarcoding_plants | Molecular | fixed | scale(gc) | 0.13 | 0.48 | 0.27 | 14.65 | 0.7923276 |
| Metabarcoding_plants | Full | fixed | Intercept | 0.08 | 0.57 | 0.15 | 8.53 | 0.8870986 |
| Metabarcoding_plants | Full | fixed | logit(composition) | 1.28 | 0.25 | 5.16 | 48.56 | 0.0000045 |
| Metabarcoding_plants | Full | fixed | scale(corr_adl) | -0.48 | 0.42 | -1.15 | 9.55 | 0.2785550 |
| Metabarcoding_plants | Full | fixed | scale(corr_aia) | 0.31 | 0.84 | 0.37 | 7.89 | 0.7219398 |
| Metabarcoding_plants | Full | fixed | scale(length) | -1.13 | 0.65 | -1.74 | 12.20 | 0.1066382 |
| Metabarcoding_plants | Full | fixed | scale(copies) | 0.21 | 0.52 | 0.40 | 9.94 | 0.7009648 |
| Metabarcoding_plants | Full | fixed | scale(gc) | 0.18 | 0.52 | 0.35 | 12.39 | 0.7353709 |
| Metagenomics_deer | Composition | fixed | Intercept | 1.22 | 0.97 | 1.26 | 20.87 | 0.2219792 |
| Metagenomics_deer | Composition | fixed | logit(composition) | 1.78 | 0.35 | 5.12 | 66.46 | 0.0000028 |
| Metagenomics_deer | Digestion | fixed | Intercept | -0.12 | 0.76 | -0.16 | 13.12 | 0.8764904 |
| Metagenomics_deer | Digestion | fixed | logit(composition) | 1.64 | 0.31 | 5.29 | 47.28 | 0.0000031 |
| Metagenomics_deer | Digestion | fixed | scale(corr_adl) | 2.21 | 0.55 | 4.03 | 12.33 | 0.0015829 |
| Metagenomics_deer | Digestion | fixed | scale(corr_aia) | -2.48 | 0.94 | -2.63 | 11.42 | 0.0227222 |
| Metagenomics_deer | Molecular | fixed | Intercept | 1.11 | 1.00 | 1.11 | 16.62 | 0.2813886 |
| Metagenomics_deer | Molecular | fixed | logit(composition) | 1.81 | 0.35 | 5.13 | 62.29 | 0.0000031 |
| Metagenomics_deer | Molecular | fixed | scale(length) | 0.62 | 0.95 | 0.65 | 12.45 | 0.5268885 |
| Metagenomics_deer | Molecular | fixed | scale(copies) | 0.55 | 0.92 | 0.59 | 12.49 | 0.5640771 |
| Metagenomics_deer | Molecular | fixed | scale(gc) | -0.82 | 0.77 | -1.07 | 12.45 | 0.3068429 |
| Metagenomics_deer | Full | fixed | Intercept | 0.00 | 0.85 | 0.00 | 10.45 | 0.9998811 |
| Metagenomics_deer | Full | fixed | logit(composition) | 1.73 | 0.33 | 5.26 | 47.53 | 0.0000034 |
| Metagenomics_deer | Full | fixed | scale(corr_adl) | 2.39 | 0.73 | 3.27 | 9.83 | 0.0085521 |
| Metagenomics_deer | Full | fixed | scale(corr_aia) | -2.24 | 1.27 | -1.77 | 9.43 | 0.1090581 |
| Metagenomics_deer | Full | fixed | scale(length) | 0.24 | 0.81 | 0.29 | 10.25 | 0.7766843 |
| Metagenomics_deer | Full | fixed | scale(copies) | 0.22 | 0.70 | 0.31 | 9.81 | 0.7635578 |
| Metagenomics_deer | Full | fixed | scale(gc) | 0.34 | 0.70 | 0.48 | 9.97 | 0.6394779 |
| Metagenomics_plants | Composition | fixed | Intercept | 0.21 | 0.48 | 0.43 | 22.67 | 0.6678351 |
| Metagenomics_plants | Composition | fixed | logit(composition) | 1.47 | 0.18 | 8.10 | 63.15 | 0.0000000 |
| Metagenomics_plants | Digestion | fixed | Intercept | 0.28 | 0.57 | 0.50 | 15.98 | 0.6257041 |
| Metagenomics_plants | Digestion | fixed | logit(composition) | 1.45 | 0.19 | 7.61 | 64.90 | 0.0000000 |
| Metagenomics_plants | Digestion | fixed | scale(corr_adl) | -0.11 | 0.41 | -0.28 | 15.34 | 0.7863061 |
| Metagenomics_plants | Digestion | fixed | scale(corr_aia) | 0.23 | 0.72 | 0.32 | 14.35 | 0.7570053 |
| Metagenomics_plants | Molecular | fixed | Intercept | 0.18 | 0.53 | 0.35 | 18.70 | 0.7334147 |
| Metagenomics_plants | Molecular | fixed | logit(composition) | 1.46 | 0.19 | 7.72 | 61.73 | 0.0000000 |
| Metagenomics_plants | Molecular | fixed | scale(length) | 0.03 | 0.50 | 0.05 | 14.47 | 0.9577286 |
| Metagenomics_plants | Molecular | fixed | scale(copies) | 0.04 | 0.48 | 0.07 | 14.59 | 0.9428562 |
| Metagenomics_plants | Molecular | fixed | scale(gc) | 0.14 | 0.41 | 0.35 | 14.57 | 0.7332628 |
| Metagenomics_plants | Full | fixed | Intercept | 0.28 | 0.63 | 0.45 | 12.95 | 0.6620063 |
| Metagenomics_plants | Full | fixed | logit(composition) | 1.43 | 0.20 | 7.19 | 63.29 | 0.0000000 |
| Metagenomics_plants | Full | fixed | scale(corr_adl) | -0.05 | 0.55 | -0.09 | 12.64 | 0.9292065 |
| Metagenomics_plants | Full | fixed | scale(corr_aia) | 0.40 | 0.96 | 0.42 | 12.14 | 0.6841121 |
| Metagenomics_plants | Full | fixed | scale(length) | 0.14 | 0.61 | 0.24 | 13.08 | 0.8161047 |
| Metagenomics_plants | Full | fixed | scale(copies) | 0.07 | 0.53 | 0.13 | 12.66 | 0.9002972 |
| Metagenomics_plants | Full | fixed | scale(gc) | 0.13 | 0.53 | 0.24 | 12.84 | 0.8150274 |
| Hybridization_deer | Composition | fixed | Intercept | 0.56 | 0.80 | 0.69 | 22.07 | 0.4947767 |
| Hybridization_deer | Composition | fixed | logit(composition) | 1.29 | 0.31 | 4.19 | 62.75 | 0.0000895 |
| Hybridization_deer | Digestion | fixed | Intercept | -0.34 | 0.66 | -0.52 | 14.86 | 0.6092759 |
| Hybridization_deer | Digestion | fixed | logit(composition) | 1.27 | 0.28 | 4.59 | 46.82 | 0.0000328 |
| Hybridization_deer | Digestion | fixed | scale(corr_adl) | 1.81 | 0.47 | 3.82 | 14.21 | 0.0018242 |
| Hybridization_deer | Digestion | fixed | scale(corr_aia) | -1.87 | 0.81 | -2.31 | 13.00 | 0.0382042 |
| Hybridization_deer | Molecular | fixed | Intercept | 0.33 | 0.78 | 0.43 | 17.28 | 0.6740654 |
| Hybridization_deer | Molecular | fixed | logit(composition) | 1.24 | 0.30 | 4.07 | 54.27 | 0.0001529 |
| Hybridization_deer | Molecular | fixed | scale(length) | 1.09 | 0.72 | 1.51 | 12.94 | 0.1562555 |
| Hybridization_deer | Molecular | fixed | scale(copies) | -0.03 | 0.70 | -0.05 | 12.96 | 0.9620580 |
| Hybridization_deer | Molecular | fixed | scale(gc) | -0.83 | 0.59 | -1.41 | 12.95 | 0.1828626 |
| Hybridization_deer | Full | fixed | Intercept | -0.31 | 0.67 | -0.46 | 10.91 | 0.6525707 |
| Hybridization_deer | Full | fixed | logit(composition) | 1.20 | 0.28 | 4.27 | 39.63 | 0.0001172 |
| Hybridization_deer | Full | fixed | scale(corr_adl) | 1.88 | 0.57 | 3.31 | 10.67 | 0.0072708 |
| Hybridization_deer | Full | fixed | scale(corr_aia) | -1.20 | 0.98 | -1.22 | 9.82 | 0.2522920 |
| Hybridization_deer | Full | fixed | scale(length) | 0.94 | 0.63 | 1.49 | 11.19 | 0.1629451 |
| Hybridization_deer | Full | fixed | scale(copies) | -0.23 | 0.55 | -0.42 | 10.64 | 0.6828168 |
| Hybridization_deer | Full | fixed | scale(gc) | 0.11 | 0.54 | 0.20 | 10.88 | 0.8451193 |
| Hybridization_plants | Composition | fixed | Intercept | 0.26 | 0.56 | 0.46 | 22.60 | 0.6473023 |
| Hybridization_plants | Composition | fixed | logit(composition) | 1.39 | 0.20 | 6.98 | 67.38 | 0.0000000 |
| Hybridization_plants | Digestion | fixed | Intercept | 0.23 | 0.67 | 0.35 | 15.99 | 0.7336500 |
| Hybridization_plants | Digestion | fixed | logit(composition) | 1.38 | 0.21 | 6.66 | 67.28 | 0.0000000 |
| Hybridization_plants | Digestion | fixed | scale(corr_adl) | 0.05 | 0.49 | 0.09 | 15.15 | 0.9268420 |
| Hybridization_plants | Digestion | fixed | scale(corr_aia) | -0.03 | 0.85 | -0.03 | 14.46 | 0.9750147 |
| Hybridization_plants | Molecular | fixed | Intercept | 0.16 | 0.61 | 0.26 | 18.60 | 0.7988082 |
| Hybridization_plants | Molecular | fixed | logit(composition) | 1.38 | 0.21 | 6.71 | 64.88 | 0.0000000 |
| Hybridization_plants | Molecular | fixed | scale(length) | 0.41 | 0.59 | 0.71 | 14.42 | 0.4908517 |
| Hybridization_plants | Molecular | fixed | scale(copies) | -0.04 | 0.56 | -0.08 | 14.53 | 0.9408702 |
| Hybridization_plants | Molecular | fixed | scale(gc) | 0.16 | 0.48 | 0.33 | 14.49 | 0.7426435 |
| Hybridization_plants | Full | fixed | Intercept | 0.25 | 0.73 | 0.35 | 12.99 | 0.7351467 |
| Hybridization_plants | Full | fixed | logit(composition) | 1.35 | 0.21 | 6.30 | 64.62 | 0.0000000 |
| Hybridization_plants | Full | fixed | scale(corr_adl) | 0.20 | 0.63 | 0.31 | 12.58 | 0.7589546 |
| Hybridization_plants | Full | fixed | scale(corr_aia) | 0.46 | 1.10 | 0.41 | 12.24 | 0.6875514 |
| Hybridization_plants | Full | fixed | scale(length) | 0.57 | 0.70 | 0.82 | 12.99 | 0.4290781 |
| Hybridization_plants | Full | fixed | scale(copies) | -0.01 | 0.61 | -0.02 | 12.60 | 0.9814445 |
| Hybridization_plants | Full | fixed | scale(gc) | 0.28 | 0.61 | 0.46 | 12.76 | 0.6556083 |

Table S5. Fixed-effect coefficients from linear mixed-effects models using logit-transformed Relative Genome Coverage (RGC) as the response variable. Models were fit separately by molecular method and sample type, with plant species and pellet type as a random intercept. Covariate descriptions and model structures (Consumption, Digestion, Molecular, and Full) are provided in the Table S4 caption. All continuous covariates were standardized (mean = 0, SD = 1).

| **dataset_name** | **model_name** | **effect** | **term** | **estimate** | **std.error** | **statistic** | **df** | **p.value** |
| --- | --- | --- | --- | --- | --- | --- | --- | --- |
| Metagenomics_deer | Composition | fixed | Intercept | 0.22 | 0.68 | 0.33 | 21.97 | 0.7460161 |
| Metagenomics_deer | Composition | fixed | logit(composition) | 0.92 | 0.27 | 3.38 | 56.94 | 0.0013051 |
| Metagenomics_deer | Digestion | fixed | Intercept | -0.41 | 0.58 | -0.70 | 14.61 | 0.4919235 |
| Metagenomics_deer | Digestion | fixed | logit(composition) | 0.93 | 0.25 | 3.73 | 43.65 | 0.0005550 |
| Metagenomics_deer | Digestion | fixed | scale(corr_adl) | 1.44 | 0.41 | 3.48 | 14.29 | 0.0035626 |
| Metagenomics_deer | Digestion | fixed | scale(corr_aia) | -1.34 | 0.71 | -1.90 | 12.78 | 0.0806011 |
| Metagenomics_deer | Molecular | fixed | Intercept | 0.17 | 0.71 | 0.24 | 17.92 | 0.8156183 |
| Metagenomics_deer | Molecular | fixed | logit(composition) | 0.95 | 0.28 | 3.39 | 53.04 | 0.0013159 |
| Metagenomics_deer | Molecular | fixed | scale(length) | 0.41 | 0.65 | 0.63 | 13.51 | 0.5393156 |
| Metagenomics_deer | Molecular | fixed | scale(copies) | 0.28 | 0.63 | 0.44 | 13.52 | 0.6665025 |
| Metagenomics_deer | Molecular | fixed | scale(gc) | -0.47 | 0.53 | -0.88 | 13.52 | 0.3932272 |
| Metagenomics_deer | Full | fixed | Intercept | -0.36 | 0.63 | -0.57 | 11.76 | 0.5785316 |
| Metagenomics_deer | Full | fixed | logit(composition) | 0.96 | 0.26 | 3.64 | 41.60 | 0.0007416 |
| Metagenomics_deer | Full | fixed | scale(corr_adl) | 1.64 | 0.54 | 3.05 | 11.46 | 0.0105758 |
| Metagenomics_deer | Full | fixed | scale(corr_aia) | -1.11 | 0.93 | -1.20 | 10.60 | 0.2559954 |
| Metagenomics_deer | Full | fixed | scale(length) | 0.26 | 0.60 | 0.44 | 12.00 | 0.6700868 |
| Metagenomics_deer | Full | fixed | scale(copies) | 0.11 | 0.52 | 0.21 | 11.42 | 0.8369083 |
| Metagenomics_deer | Full | fixed | scale(gc) | 0.35 | 0.51 | 0.68 | 11.67 | 0.5079201 |
| Metagenomics_plants | Composition | fixed | Intercept | -1.03 | 0.44 | -2.36 | 18.14 | 0.0297907 |
| Metagenomics_plants | Composition | fixed | logit(composition) | 0.27 | 0.09 | 2.90 | 60.80 | 0.0051614 |
| Metagenomics_plants | Digestion | fixed | Intercept | -0.59 | 0.52 | -1.14 | 14.70 | 0.2706424 |
| Metagenomics_plants | Digestion | fixed | logit(composition) | 0.25 | 0.09 | 2.73 | 59.93 | 0.0083149 |
| Metagenomics_plants | Digestion | fixed | scale(corr_adl) | -0.17 | 0.39 | -0.44 | 14.11 | 0.6698146 |
| Metagenomics_plants | Digestion | fixed | scale(corr_aia) | 1.05 | 0.67 | 1.56 | 14.04 | 0.1411883 |
| Metagenomics_plants | Molecular | fixed | Intercept | -1.03 | 0.49 | -2.10 | 15.06 | 0.0534718 |
| Metagenomics_plants | Molecular | fixed | logit(composition) | 0.25 | 0.09 | 2.67 | 58.97 | 0.0097824 |
| Metagenomics_plants | Molecular | fixed | scale(length) | -0.08 | 0.50 | -0.16 | 13.40 | 0.8760656 |
| Metagenomics_plants | Molecular | fixed | scale(copies) | -0.11 | 0.48 | -0.22 | 13.43 | 0.8258980 |
| Metagenomics_plants | Molecular | fixed | scale(gc) | 0.00 | 0.41 | 0.00 | 13.41 | 0.9962598 |
| Metagenomics_plants | Full | fixed | Intercept | -0.57 | 0.58 | -0.97 | 12.14 | 0.3528435 |
| Metagenomics_plants | Full | fixed | logit(composition) | 0.23 | 0.09 | 2.48 | 57.93 | 0.0159717 |
| Metagenomics_plants | Full | fixed | scale(corr_adl) | -0.21 | 0.51 | -0.41 | 11.87 | 0.6911613 |
| Metagenomics_plants | Full | fixed | scale(corr_aia) | 1.32 | 0.90 | 1.47 | 11.87 | 0.1664481 |
| Metagenomics_plants | Full | fixed | scale(length) | 0.28 | 0.56 | 0.49 | 12.00 | 0.6323855 |
| Metagenomics_plants | Full | fixed | scale(copies) | 0.02 | 0.49 | 0.05 | 11.88 | 0.9627535 |
| Metagenomics_plants | Full | fixed | scale(gc) | -0.06 | 0.49 | -0.13 | 11.91 | 0.9020896 |
| Hybridization_deer | Composition | fixed | Intercept | -0.30 | 0.24 | -1.27 | 21.30 | 0.2177290 |
| Hybridization_deer | Composition | fixed | logit(composition) | 0.58 | 0.11 | 5.47 | 43.95 | 0.0000020 |
| Hybridization_deer | Digestion | fixed | Intercept | -0.34 | 0.26 | -1.31 | 15.70 | 0.2088387 |
| Hybridization_deer | Digestion | fixed | logit(composition) | 0.60 | 0.11 | 5.34 | 46.77 | 0.0000027 |
| Hybridization_deer | Digestion | fixed | scale(corr_adl) | 0.22 | 0.19 | 1.15 | 15.13 | 0.2694828 |
| Hybridization_deer | Digestion | fixed | scale(corr_aia) | -0.12 | 0.33 | -0.38 | 13.76 | 0.7102660 |
| Hybridization_deer | Molecular | fixed | Intercept | -0.34 | 0.25 | -1.37 | 17.97 | 0.1866407 |
| Hybridization_deer | Molecular | fixed | logit(composition) | 0.59 | 0.11 | 5.37 | 40.76 | 0.0000034 |
| Hybridization_deer | Molecular | fixed | scale(length) | 0.28 | 0.22 | 1.22 | 14.36 | 0.2405277 |
| Hybridization_deer | Molecular | fixed | scale(copies) | -0.01 | 0.22 | -0.03 | 14.31 | 0.9739576 |
| Hybridization_deer | Molecular | fixed | scale(gc) | 0.01 | 0.18 | 0.03 | 14.43 | 0.9727597 |
| Hybridization_deer | Full | fixed | Intercept | -0.33 | 0.26 | -1.26 | 12.01 | 0.2310313 |
| Hybridization_deer | Full | fixed | logit(composition) | 0.58 | 0.11 | 5.11 | 39.53 | 0.0000087 |
| Hybridization_deer | Full | fixed | scale(corr_adl) | 0.32 | 0.22 | 1.42 | 11.90 | 0.1802624 |
| Hybridization_deer | Full | fixed | scale(corr_aia) | 0.18 | 0.39 | 0.47 | 10.83 | 0.6504559 |
| Hybridization_deer | Full | fixed | scale(length) | 0.36 | 0.25 | 1.47 | 12.49 | 0.1666731 |
| Hybridization_deer | Full | fixed | scale(copies) | 0.00 | 0.22 | -0.02 | 11.86 | 0.9818919 |
| Hybridization_deer | Full | fixed | scale(gc) | 0.18 | 0.21 | 0.84 | 12.15 | 0.4180076 |
| Hybridization_plants | Composition | fixed | Intercept | -0.49 | 0.29 | -1.68 | 19.46 | 0.1087754 |
| Hybridization_plants | Composition | fixed | logit(composition) | 0.47 | 0.08 | 5.73 | 68.40 | 0.0000003 |
| Hybridization_plants | Digestion | fixed | Intercept | -0.21 | 0.33 | -0.62 | 14.85 | 0.5436286 |
| Hybridization_plants | Digestion | fixed | logit(composition) | 0.45 | 0.08 | 5.47 | 66.57 | 0.0000007 |
| Hybridization_plants | Digestion | fixed | scale(corr_adl) | -0.21 | 0.24 | -0.85 | 13.99 | 0.4091417 |
| Hybridization_plants | Digestion | fixed | scale(corr_aia) | 0.68 | 0.43 | 1.61 | 13.74 | 0.1311289 |
| Hybridization_plants | Molecular | fixed | Intercept | -0.48 | 0.32 | -1.51 | 15.76 | 0.1505707 |
| Hybridization_plants | Molecular | fixed | logit(composition) | 0.46 | 0.08 | 5.48 | 65.45 | 0.0000007 |
| Hybridization_plants | Molecular | fixed | scale(length) | -0.10 | 0.32 | -0.31 | 12.87 | 0.7601421 |
| Hybridization_plants | Molecular | fixed | scale(copies) | -0.08 | 0.30 | -0.26 | 12.93 | 0.7993246 |
| Hybridization_plants | Molecular | fixed | scale(gc) | 0.17 | 0.26 | 0.66 | 12.89 | 0.5183199 |
| Hybridization_plants | Full | fixed | Intercept | -0.22 | 0.37 | -0.59 | 12.06 | 0.5689712 |
| Hybridization_plants | Full | fixed | logit(composition) | 0.43 | 0.08 | 5.15 | 63.14 | 0.0000028 |
| Hybridization_plants | Full | fixed | scale(corr_adl) | -0.14 | 0.32 | -0.43 | 11.65 | 0.6718634 |
| Hybridization_plants | Full | fixed | scale(corr_aia) | 0.79 | 0.57 | 1.39 | 11.57 | 0.1914881 |
| Hybridization_plants | Full | fixed | scale(length) | 0.12 | 0.36 | 0.33 | 11.90 | 0.7505393 |
| Hybridization_plants | Full | fixed | scale(copies) | 0.00 | 0.31 | -0.01 | 11.66 | 0.9941958 |
| Hybridization_plants | Full | fixed | scale(gc) | 0.12 | 0.31 | 0.39 | 11.74 | 0.7033048 |

Table S6. Fixed-effect coefficients from linear mixed-effects models using logit-transformed Relative Read Abundance (RRA) as the response variable, with covariates in their original (unstandardized) units. Model structure and covariate definitions follow those described in Table S4.

| **dataset_name** | **model_name** | **effect** | **term** | **estimate** | **std.error** | **statistic** | **df** | **p.value** |
| --- | --- | --- | --- | --- | --- | --- | --- | --- |
| Metabarcoding_deer | Composition | fixed | Intercept | 0.74 | 0.85 | 0.86 | 18.60 | 0.3988386 |
| Metabarcoding_deer | Composition | fixed | logit(composition) | 1.41 | 0.35 | 4.07 | 51.63 | 0.0001594 |
| Metabarcoding_deer | Digestion | fixed | Intercept | -0.54 | 1.18 | -0.46 | 14.16 | 0.6508980 |
| Metabarcoding_deer | Digestion | fixed | logit(composition) | 1.46 | 0.29 | 4.98 | 33.72 | 0.0000185 |
| Metabarcoding_deer | Digestion | fixed | corr_adl | 0.45 | 0.11 | 4.02 | 12.99 | 0.0014608 |
| Metabarcoding_deer | Digestion | fixed | corr_aia | -1.48 | 0.54 | -2.73 | 11.23 | 0.0192192 |
| Metabarcoding_deer | Molecular | fixed | Intercept | -13.69 | 11.47 | -1.19 | 14.82 | 0.2512411 |
| Metabarcoding_deer | Molecular | fixed | logit(composition) | 1.62 | 0.38 | 4.23 | 56.50 | 0.0000864 |
| Metabarcoding_deer | Molecular | fixed | length | 0.13 | 0.09 | 1.41 | 13.72 | 0.1813018 |
| Metabarcoding_deer | Molecular | fixed | copies | 0.00 | 0.00 | -0.02 | 12.48 | 0.9866613 |
| Metabarcoding_deer | Molecular | fixed | gc | 0.09 | 0.18 | 0.50 | 14.63 | 0.6220471 |
| Metabarcoding_deer | Full | fixed | Intercept | -8.14 | 9.45 | -0.86 | 11.88 | 0.4059440 |
| Metabarcoding_deer | Full | fixed | logit(composition) | 1.55 | 0.33 | 4.67 | 36.52 | 0.0000401 |
| Metabarcoding_deer | Full | fixed | corr_adl | 0.45 | 0.12 | 3.89 | 10.08 | 0.0029439 |
| Metabarcoding_deer | Full | fixed | corr_aia | -1.20 | 0.66 | -1.82 | 8.66 | 0.1036823 |
| Metabarcoding_deer | Full | fixed | length | 0.09 | 0.07 | 1.38 | 11.32 | 0.1935062 |
| Metabarcoding_deer | Full | fixed | copies | 0.00 | 0.00 | -0.69 | 10.11 | 0.5055865 |
| Metabarcoding_deer | Full | fixed | gc | -0.01 | 0.13 | -0.10 | 12.00 | 0.9220474 |
| Metabarcoding_plants | Composition | fixed | Intercept | -0.19 | 0.54 | -0.36 | 16.37 | 0.7257994 |
| Metabarcoding_plants | Composition | fixed | logit(composition) | 1.39 | 0.22 | 6.33 | 43.75 | 0.0000001 |
| Metabarcoding_plants | Digestion | fixed | Intercept | -0.32 | 1.08 | -0.30 | 13.75 | 0.7711428 |
| Metabarcoding_plants | Digestion | fixed | logit(composition) | 1.30 | 0.23 | 5.71 | 47.10 | 0.0000007 |
| Metabarcoding_plants | Digestion | fixed | corr_adl | -0.11 | 0.10 | -1.09 | 12.68 | 0.2947276 |
| Metabarcoding_plants | Digestion | fixed | corr_aia | 0.64 | 0.50 | 1.27 | 10.92 | 0.2307509 |
| Metabarcoding_plants | Molecular | fixed | Intercept | 7.55 | 6.84 | 1.10 | 15.62 | 0.2870060 |
| Metabarcoding_plants | Molecular | fixed | logit(composition) | 1.31 | 0.24 | 5.38 | 51.37 | 0.0000019 |
| Metabarcoding_plants | Molecular | fixed | length | -0.11 | 0.05 | -1.99 | 14.15 | 0.0664076 |
| Metabarcoding_plants | Molecular | fixed | copies | 0.00 | 0.00 | 0.22 | 11.87 | 0.8293293 |
| Metabarcoding_plants | Molecular | fixed | gc | 0.03 | 0.10 | 0.27 | 14.65 | 0.7923276 |
| Metabarcoding_plants | Full | fixed | Intercept | 7.45 | 8.29 | 0.90 | 13.01 | 0.3855331 |
| Metabarcoding_plants | Full | fixed | logit(composition) | 1.28 | 0.25 | 5.16 | 48.56 | 0.0000045 |
| Metabarcoding_plants | Full | fixed | corr_adl | -0.11 | 0.10 | -1.15 | 9.55 | 0.2785550 |
| Metabarcoding_plants | Full | fixed | corr_aia | 0.21 | 0.56 | 0.37 | 7.89 | 0.7219398 |
| Metabarcoding_plants | Full | fixed | length | -0.10 | 0.06 | -1.74 | 12.20 | 0.1066382 |
| Metabarcoding_plants | Full | fixed | copies | 0.00 | 0.00 | 0.40 | 9.94 | 0.7009648 |
| Metabarcoding_plants | Full | fixed | gc | 0.04 | 0.11 | 0.35 | 12.39 | 0.7353710 |
| Metagenomics_deer | Composition | fixed | Intercept | 1.22 | 0.97 | 1.26 | 20.87 | 0.2219792 |
| Metagenomics_deer | Composition | fixed | logit(composition) | 1.78 | 0.35 | 5.12 | 66.46 | 0.0000028 |
| Metagenomics_deer | Digestion | fixed | Intercept | -0.68 | 1.36 | -0.50 | 14.61 | 0.6266612 |
| Metagenomics_deer | Digestion | fixed | logit(composition) | 1.64 | 0.31 | 5.29 | 47.28 | 0.0000031 |
| Metagenomics_deer | Digestion | fixed | corr_adl | 0.53 | 0.13 | 4.03 | 12.33 | 0.0015829 |
| Metagenomics_deer | Digestion | fixed | corr_aia | -1.72 | 0.65 | -2.63 | 11.42 | 0.0227222 |
| Metagenomics_deer | Molecular | fixed | Intercept | 28.18 | 37.65 | 0.75 | 12.32 | 0.4682912 |
| Metagenomics_deer | Molecular | fixed | logit(composition) | 1.81 | 0.35 | 5.13 | 62.29 | 0.0000031 |
| Metagenomics_deer | Molecular | fixed | length | 0.00 | 0.00 | 0.65 | 12.45 | 0.5268885 |
| Metagenomics_deer | Molecular | fixed | copies | 0.00 | 0.00 | 0.59 | 12.49 | 0.5640771 |
| Metagenomics_deer | Molecular | fixed | gc | -1.01 | 0.95 | -1.07 | 12.45 | 0.3068428 |
| Metagenomics_deer | Full | fixed | Intercept | -20.48 | 34.96 | -0.59 | 9.88 | 0.5710603 |
| Metagenomics_deer | Full | fixed | logit(composition) | 1.73 | 0.33 | 5.26 | 47.53 | 0.0000034 |
| Metagenomics_deer | Full | fixed | corr_adl | 0.58 | 0.18 | 3.27 | 9.83 | 0.0085521 |
| Metagenomics_deer | Full | fixed | corr_aia | -1.55 | 0.88 | -1.77 | 9.43 | 0.1090581 |
| Metagenomics_deer | Full | fixed | length | 0.00 | 0.00 | 0.29 | 10.25 | 0.7766843 |
| Metagenomics_deer | Full | fixed | copies | 0.00 | 0.00 | 0.31 | 9.81 | 0.7635578 |
| Metagenomics_deer | Full | fixed | gc | 0.41 | 0.85 | 0.48 | 9.97 | 0.6394779 |
| Metagenomics_plants | Composition | fixed | Intercept | 0.21 | 0.48 | 0.43 | 22.67 | 0.6678351 |
| Metagenomics_plants | Composition | fixed | logit(composition) | 1.47 | 0.18 | 8.10 | 63.15 | 0.0000000 |
| Metagenomics_plants | Digestion | fixed | Intercept | 0.19 | 1.02 | 0.18 | 18.08 | 0.8574452 |
| Metagenomics_plants | Digestion | fixed | logit(composition) | 1.45 | 0.19 | 7.61 | 64.90 | 0.0000000 |
| Metagenomics_plants | Digestion | fixed | corr_adl | -0.03 | 0.10 | -0.28 | 15.34 | 0.7863061 |
| Metagenomics_plants | Digestion | fixed | corr_aia | 0.16 | 0.49 | 0.32 | 14.35 | 0.7570053 |
| Metagenomics_plants | Molecular | fixed | Intercept | -6.77 | 19.94 | -0.34 | 14.41 | 0.7390677 |
| Metagenomics_plants | Molecular | fixed | logit(composition) | 1.46 | 0.19 | 7.72 | 61.73 | 0.0000000 |
| Metagenomics_plants | Molecular | fixed | length | 0.00 | 0.00 | 0.05 | 14.47 | 0.9577286 |
| Metagenomics_plants | Molecular | fixed | copies | 0.00 | 0.00 | 0.07 | 14.59 | 0.9428562 |
| Metagenomics_plants | Molecular | fixed | gc | 0.17 | 0.50 | 0.35 | 14.57 | 0.7332628 |
| Metagenomics_plants | Full | fixed | Intercept | -8.25 | 26.41 | -0.31 | 12.75 | 0.7597817 |
| Metagenomics_plants | Full | fixed | logit(composition) | 1.43 | 0.20 | 7.19 | 63.29 | 0.0000000 |
| Metagenomics_plants | Full | fixed | corr_adl | -0.01 | 0.13 | -0.09 | 12.64 | 0.9292065 |
| Metagenomics_plants | Full | fixed | corr_aia | 0.27 | 0.66 | 0.42 | 12.14 | 0.6841121 |
| Metagenomics_plants | Full | fixed | length | 0.00 | 0.00 | 0.24 | 13.08 | 0.8161047 |
| Metagenomics_plants | Full | fixed | copies | 0.00 | 0.00 | 0.13 | 12.66 | 0.9002972 |
| Metagenomics_plants | Full | fixed | gc | 0.15 | 0.64 | 0.24 | 12.84 | 0.8150274 |
| Hybridization_deer | Composition | fixed | Intercept | 0.56 | 0.80 | 0.69 | 22.07 | 0.4947767 |
| Hybridization_deer | Composition | fixed | logit(composition) | 1.29 | 0.31 | 4.19 | 62.75 | 0.0000895 |
| Hybridization_deer | Digestion | fixed | Intercept | -1.00 | 1.18 | -0.84 | 16.70 | 0.4107769 |
| Hybridization_deer | Digestion | fixed | logit(composition) | 1.27 | 0.28 | 4.59 | 46.82 | 0.0000328 |
| Hybridization_deer | Digestion | fixed | corr_adl | 0.44 | 0.11 | 3.82 | 14.21 | 0.0018242 |
| Hybridization_deer | Digestion | fixed | corr_aia | -1.30 | 0.56 | -2.31 | 13.00 | 0.0382042 |
| Hybridization_deer | Molecular | fixed | Intercept | 20.45 | 28.61 | 0.71 | 12.77 | 0.4875273 |
| Hybridization_deer | Molecular | fixed | logit(composition) | 1.24 | 0.30 | 4.07 | 54.27 | 0.0001529 |
| Hybridization_deer | Molecular | fixed | length | 0.00 | 0.00 | 1.51 | 12.94 | 0.1562555 |
| Hybridization_deer | Molecular | fixed | copies | 0.00 | 0.00 | -0.05 | 12.96 | 0.9620580 |
| Hybridization_deer | Molecular | fixed | gc | -1.01 | 0.72 | -1.41 | 12.95 | 0.1828626 |
| Hybridization_deer | Full | fixed | Intercept | -22.01 | 27.27 | -0.81 | 10.76 | 0.4370610 |
| Hybridization_deer | Full | fixed | logit(composition) | 1.20 | 0.28 | 4.27 | 39.63 | 0.0001172 |
| Hybridization_deer | Full | fixed | corr_adl | 0.45 | 0.14 | 3.31 | 10.67 | 0.0072708 |
| Hybridization_deer | Full | fixed | corr_aia | -0.83 | 0.68 | -1.22 | 9.82 | 0.2522920 |
| Hybridization_deer | Full | fixed | length | 0.00 | 0.00 | 1.49 | 11.19 | 0.1629451 |
| Hybridization_deer | Full | fixed | copies | 0.00 | 0.00 | -0.42 | 10.64 | 0.6828168 |
| Hybridization_deer | Full | fixed | gc | 0.13 | 0.67 | 0.20 | 10.88 | 0.8451193 |
| Hybridization_plants | Composition | fixed | Intercept | 0.26 | 0.56 | 0.46 | 22.60 | 0.6473023 |
| Hybridization_plants | Composition | fixed | logit(composition) | 1.39 | 0.20 | 6.98 | 67.38 | 0.0000000 |
| Hybridization_plants | Digestion | fixed | Intercept | 0.19 | 1.19 | 0.16 | 17.65 | 0.8745441 |
| Hybridization_plants | Digestion | fixed | logit(composition) | 1.38 | 0.21 | 6.66 | 67.28 | 0.0000000 |
| Hybridization_plants | Digestion | fixed | corr_adl | 0.01 | 0.12 | 0.09 | 15.15 | 0.9268420 |
| Hybridization_plants | Digestion | fixed | corr_aia | -0.02 | 0.59 | -0.03 | 14.46 | 0.9750147 |
| Hybridization_plants | Molecular | fixed | Intercept | -13.74 | 23.17 | -0.59 | 14.35 | 0.5622952 |
| Hybridization_plants | Molecular | fixed | logit(composition) | 1.38 | 0.21 | 6.71 | 64.88 | 0.0000000 |
| Hybridization_plants | Molecular | fixed | length | 0.00 | 0.00 | 0.71 | 14.42 | 0.4908518 |
| Hybridization_plants | Molecular | fixed | copies | 0.00 | 0.00 | -0.08 | 14.53 | 0.9408702 |
| Hybridization_plants | Molecular | fixed | gc | 0.20 | 0.58 | 0.33 | 14.49 | 0.7426436 |
| Hybridization_plants | Full | fixed | Intercept | -22.52 | 30.48 | -0.74 | 12.68 | 0.4734964 |
| Hybridization_plants | Full | fixed | logit(composition) | 1.35 | 0.21 | 6.30 | 64.62 | 0.0000000 |
| Hybridization_plants | Full | fixed | corr_adl | 0.05 | 0.15 | 0.31 | 12.58 | 0.7589547 |
| Hybridization_plants | Full | fixed | corr_aia | 0.31 | 0.76 | 0.41 | 12.24 | 0.6875514 |
| Hybridization_plants | Full | fixed | length | 0.00 | 0.00 | 0.82 | 12.99 | 0.4290782 |
| Hybridization_plants | Full | fixed | copies | 0.00 | 0.00 | -0.02 | 12.60 | 0.9814445 |
| Hybridization_plants | Full | fixed | gc | 0.34 | 0.74 | 0.46 | 12.76 | 0.6556084 |

Table S7. Fixed-effect coefficients from linear mixed-effects models using logit-transformed Relative Genome Coverage (RGC) as the response variable, with covariates in their original (unstandardized) units. Model structure and covariate definitions follow those described in Table S4.

| **dataset_name** | **model_name** | **effect** | **term** | **estimate** | **std.error** | **statistic** | **df** | **p.value** |
| --- | --- | --- | --- | --- | --- | --- | --- | --- |
| Metagenomics_deer | Composition | fixed | Intercept | 0.22 | 0.68 | 0.33 | 21.97 | 0.7460161 |
| Metagenomics_deer | Composition | fixed | logit(composition) | 0.92 | 0.27 | 3.38 | 56.94 | 0.0013051 |
| Metagenomics_deer | Digestion | fixed | Intercept | -1.12 | 1.04 | -1.07 | 16.71 | 0.2981310 |
| Metagenomics_deer | Digestion | fixed | logit(composition) | 0.93 | 0.25 | 3.73 | 43.65 | 0.0005550 |
| Metagenomics_deer | Digestion | fixed | corr_adl | 0.35 | 0.10 | 3.48 | 14.29 | 0.0035626 |
| Metagenomics_deer | Digestion | fixed | corr_aia | -0.93 | 0.49 | -1.90 | 12.78 | 0.0806011 |
| Metagenomics_deer | Molecular | fixed | Intercept | 14.60 | 25.80 | 0.57 | 13.33 | 0.5808025 |
| Metagenomics_deer | Molecular | fixed | logit(composition) | 0.95 | 0.28 | 3.39 | 53.04 | 0.0013159 |
| Metagenomics_deer | Molecular | fixed | length | 0.00 | 0.00 | 0.63 | 13.51 | 0.5393156 |
| Metagenomics_deer | Molecular | fixed | copies | 0.00 | 0.00 | 0.44 | 13.52 | 0.6665026 |
| Metagenomics_deer | Molecular | fixed | gc | -0.57 | 0.65 | -0.88 | 13.52 | 0.3932272 |
| Metagenomics_deer | Full | fixed | Intercept | -21.94 | 25.71 | -0.85 | 11.55 | 0.4107188 |
| Metagenomics_deer | Full | fixed | logit(composition) | 0.96 | 0.26 | 3.64 | 41.60 | 0.0007416 |
| Metagenomics_deer | Full | fixed | corr_adl | 0.39 | 0.13 | 3.05 | 11.46 | 0.0105758 |
| Metagenomics_deer | Full | fixed | corr_aia | -0.77 | 0.64 | -1.20 | 10.60 | 0.2559954 |
| Metagenomics_deer | Full | fixed | length | 0.00 | 0.00 | 0.44 | 12.00 | 0.6700868 |
| Metagenomics_deer | Full | fixed | copies | 0.00 | 0.00 | 0.21 | 11.42 | 0.8369083 |
| Metagenomics_deer | Full | fixed | gc | 0.43 | 0.63 | 0.68 | 11.67 | 0.5079201 |
| Metagenomics_plants | Composition | fixed | Intercept | -1.03 | 0.44 | -2.36 | 18.14 | 0.0297907 |
| Metagenomics_plants | Composition | fixed | logit(composition) | 0.27 | 0.09 | 2.90 | 60.80 | 0.0051614 |
| Metagenomics_plants | Digestion | fixed | Intercept | -1.65 | 0.91 | -1.83 | 15.12 | 0.0875991 |
| Metagenomics_plants | Digestion | fixed | logit(composition) | 0.25 | 0.09 | 2.73 | 59.93 | 0.0083149 |
| Metagenomics_plants | Digestion | fixed | corr_adl | -0.04 | 0.09 | -0.44 | 14.11 | 0.6698146 |
| Metagenomics_plants | Digestion | fixed | corr_aia | 0.73 | 0.47 | 1.56 | 14.04 | 0.1411883 |
| Metagenomics_plants | Molecular | fixed | Intercept | 0.26 | 19.99 | 0.01 | 13.37 | 0.9896573 |
| Metagenomics_plants | Molecular | fixed | logit(composition) | 0.25 | 0.09 | 2.67 | 58.97 | 0.0097824 |
| Metagenomics_plants | Molecular | fixed | length | 0.00 | 0.00 | -0.16 | 13.40 | 0.8760656 |
| Metagenomics_plants | Molecular | fixed | copies | 0.00 | 0.00 | -0.22 | 13.43 | 0.8258980 |
| Metagenomics_plants | Molecular | fixed | gc | 0.00 | 0.50 | 0.00 | 13.41 | 0.9962598 |
| Metagenomics_plants | Full | fixed | Intercept | -3.59 | 24.65 | -0.15 | 11.89 | 0.8866582 |
| Metagenomics_plants | Full | fixed | logit(composition) | 0.23 | 0.09 | 2.48 | 57.93 | 0.0159717 |
| Metagenomics_plants | Full | fixed | corr_adl | -0.05 | 0.12 | -0.41 | 11.87 | 0.6911614 |
| Metagenomics_plants | Full | fixed | corr_aia | 0.91 | 0.62 | 1.47 | 11.87 | 0.1664481 |
| Metagenomics_plants | Full | fixed | length | 0.00 | 0.00 | 0.49 | 12.00 | 0.6323855 |
| Metagenomics_plants | Full | fixed | copies | 0.00 | 0.00 | 0.05 | 11.88 | 0.9627535 |
| Metagenomics_plants | Full | fixed | gc | -0.08 | 0.60 | -0.13 | 11.91 | 0.9020896 |
| Hybridization_deer | Composition | fixed | Intercept | -0.30 | 0.24 | -1.27 | 21.30 | 0.2177290 |
| Hybridization_deer | Composition | fixed | logit(composition) | 0.58 | 0.11 | 5.47 | 43.95 | 0.0000020 |
| Hybridization_deer | Digestion | fixed | Intercept | -0.55 | 0.48 | -1.16 | 17.70 | 0.2601876 |
| Hybridization_deer | Digestion | fixed | logit(composition) | 0.60 | 0.11 | 5.34 | 46.77 | 0.0000027 |
| Hybridization_deer | Digestion | fixed | corr_adl | 0.05 | 0.05 | 1.15 | 15.13 | 0.2694828 |
| Hybridization_deer | Digestion | fixed | corr_aia | -0.09 | 0.23 | -0.38 | 13.76 | 0.7102660 |
| Hybridization_deer | Molecular | fixed | Intercept | -5.09 | 8.90 | -0.57 | 14.14 | 0.5759514 |
| Hybridization_deer | Molecular | fixed | logit(composition) | 0.59 | 0.11 | 5.37 | 40.76 | 0.0000034 |
| Hybridization_deer | Molecular | fixed | length | 0.00 | 0.00 | 1.22 | 14.36 | 0.2405277 |
| Hybridization_deer | Molecular | fixed | copies | 0.00 | 0.00 | -0.03 | 14.31 | 0.9739576 |
| Hybridization_deer | Molecular | fixed | gc | 0.01 | 0.22 | 0.03 | 14.43 | 0.9727597 |
| Hybridization_deer | Full | fixed | Intercept | -15.17 | 10.72 | -1.42 | 12.02 | 0.1824322 |
| Hybridization_deer | Full | fixed | logit(composition) | 0.58 | 0.11 | 5.11 | 39.53 | 0.0000087 |
| Hybridization_deer | Full | fixed | corr_adl | 0.08 | 0.05 | 1.42 | 11.90 | 0.1802625 |
| Hybridization_deer | Full | fixed | corr_aia | 0.12 | 0.27 | 0.47 | 10.83 | 0.6504560 |
| Hybridization_deer | Full | fixed | length | 0.00 | 0.00 | 1.47 | 12.49 | 0.1666732 |
| Hybridization_deer | Full | fixed | copies | 0.00 | 0.00 | -0.02 | 11.86 | 0.9818919 |
| Hybridization_deer | Full | fixed | gc | 0.22 | 0.26 | 0.84 | 12.15 | 0.4180077 |
| Hybridization_plants | Composition | fixed | Intercept | -0.49 | 0.29 | -1.68 | 19.46 | 0.1087754 |
| Hybridization_plants | Composition | fixed | logit(composition) | 0.47 | 0.08 | 5.73 | 68.40 | 0.0000003 |
| Hybridization_plants | Digestion | fixed | Intercept | -0.73 | 0.58 | -1.25 | 15.75 | 0.2278740 |
| Hybridization_plants | Digestion | fixed | logit(composition) | 0.45 | 0.08 | 5.47 | 66.57 | 0.0000007 |
| Hybridization_plants | Digestion | fixed | corr_adl | -0.05 | 0.06 | -0.85 | 13.99 | 0.4091417 |
| Hybridization_plants | Digestion | fixed | corr_aia | 0.47 | 0.29 | 1.61 | 13.74 | 0.1311289 |
| Hybridization_plants | Molecular | fixed | Intercept | -6.58 | 12.48 | -0.53 | 12.81 | 0.6072311 |
| Hybridization_plants | Molecular | fixed | logit(composition) | 0.46 | 0.08 | 5.48 | 65.45 | 0.0000007 |
| Hybridization_plants | Molecular | fixed | length | 0.00 | 0.00 | -0.31 | 12.87 | 0.7601421 |
| Hybridization_plants | Molecular | fixed | copies | 0.00 | 0.00 | -0.26 | 12.93 | 0.7993246 |
| Hybridization_plants | Molecular | fixed | gc | 0.21 | 0.31 | 0.66 | 12.89 | 0.5183199 |
| Hybridization_plants | Full | fixed | Intercept | -8.41 | 15.64 | -0.54 | 11.69 | 0.6011243 |
| Hybridization_plants | Full | fixed | logit(composition) | 0.43 | 0.08 | 5.15 | 63.14 | 0.0000028 |
| Hybridization_plants | Full | fixed | corr_adl | -0.03 | 0.08 | -0.43 | 11.65 | 0.6718633 |
| Hybridization_plants | Full | fixed | corr_aia | 0.54 | 0.39 | 1.39 | 11.57 | 0.1914881 |
| Hybridization_plants | Full | fixed | length | 0.00 | 0.00 | 0.33 | 11.90 | 0.7505393 |
| Hybridization_plants | Full | fixed | copies | 0.00 | 0.00 | -0.01 | 11.66 | 0.9941958 |
| Hybridization_plants | Full | fixed | gc | 0.15 | 0.38 | 0.39 | 11.74 | 0.7033048 |
